# Burden of chronic kidney disease on the African continent: a systematic review and meta-analysis

**DOI:** 10.1186/s12882-018-0930-5

**Published:** 2018-06-01

**Authors:** Arnaud D. Kaze, Titilayo Ilori, Bernard G. Jaar, Justin B. Echouffo-Tcheugui

**Affiliations:** 1 Division of Renal Medicine, Brigham and Women’s Hospital, Harvard Medical School, Boston, MA USA; 2 Department of Medicine, University of Maryland Medical Center Midtown Campus, Baltimore, MD USA; 3 Division of Renal Medicine, Emory University School of Medicine, Atlanta, GA USA; 4 Division of Nephrology, Department of Medicine, Johns Hopkins University School of Medicine, Baltimore, MD USA; 5 Department of Epidemiology, Johns Hopkins Bloomberg School of Public Health, Baltimore, MD USA; 6 Welch Center for Prevention, Epidemiology and Clinical Research, Johns Hopkins Medical Institutions, Baltimore, MD USA; 7 Nephrology Center of Maryland, Baltimore, MD USA; 8 Division of Endocrinology, Diabetes, and Hypertension, Brigham and Women’s Hospital, Harvard Medical School, 221 Longwood Avenue, Boston, MA 02115 USA

**Keywords:** Chronic kidney disease, Prevalence, Systematic review, Meta-analysis, Africa

## Abstract

**Background:**

Accurate contemporary data on the burden of Chronic Kidney Disease (CKD) on the African continent are lacking. We determined the prevalence of CKD in adult populations living in Africa, and variations by stage, gender, estimated Glomerular Filtration Rate (eGFR) equation, and residence.

**Methods:**

For this systematic review, we searched multiple electronic databases for original studies on CKD prevalence reported from January 1, 2000 to December 31, 2016. Two reviewers independently undertook quality assessment and data extraction. We stabilized the variance of study-specific estimates with the Freeman-Turkey single arcsine transformation and pooled the data using a random effects meta-analysis models.

**Results:**

A total of 98 studies involving 98,432 individuals were included in the final meta-analysis. The overall prevalence was 15.8% (95% CI 12.1–19.9) for CKD stages 1–5 and 4.6% (3.3–6.1) for CKD stages 3–5 in the general population. Equivalent figures were greater at 32.3% (23.4–41.8) and 13.3% (10.7–16.0) in high-risk populations (people with hypertension, diabetes, HIV). CKD prevalence was higher in studies based on the Cockcroft-Gault formula than MDRD or CKD-EPI equations; and in studies from sub-Saharan Africa compared with those from North Africa (17.7, 95% CI 13.7–22.1 vs 6.1, 95% CI 3.6–9.3, *p* < 0.001). There was substantial heterogeneity across studies (all I^2^ > 90%) and no evidence of publication bias in main analyses.

**Conclusion:**

CKD is highly prevalent across Africa, inviting efforts into prevention, early detection and control of CKD in adults living on the African continent which is particularly important in a resource limited environment.

**Trial Registration:**

Prospero Registration ID: CRD42017054445.

**Electronic supplementary material:**

The online version of this article (10.1186/s12882-018-0930-5) contains supplementary material, which is available to authorized users.

## Background

Chronic kidney disease (CKD) is a leading cause of morbidity and mortality in both developed and developing countries, with an estimated 10% of the population worldwide having CKD in 2015 [1, 2]. Studies have consistently shown that African descendants are at increased risk for CKD occurrence and progression to end-stage renal disease (ESRD) [3, 4]. Many African countries are currently undergoing rapid epidemiological transitions and are confronted with the double burden of communicable and non-communicable diseases, in part driven by the adoption of western lifestyles, changes in the built environment, and the rapid urbanization [5]. This dual burden has led to a consequential rise in the number of people affected by CKD on the African continent [6].

Given the constant rise in its risk factors in Africa [7, 8], CKD is increasingly recognized as a major public health threat, against a background of limited access to renal replacement therapy (RRT) [6]. Hence, in Africa, prevention and early detection of CKD in order to slow its progression are of paramount importance. For this purpose, a better understanding of the current prevalence of CKD in Africa is urgently needed. Although the number of reports on CKD prevalence across Africa has increased in recent years, accurate data on its exact magnitude on the continent are still lacking [6]. The only systematic review of CKD prevalence in Africa was limited to sub-Saharan countries, included studies published between 1962 and 2011, and highlighted the inability to make definitive inferences due to the poor quality of included studies [9]. We conducted a systematic review and meta-analysis of the contemporary evidence on CKD prevalence in adults living on the African continent, in order to establish baseline figures against which future trends can be monitored.

## Methods

We used a prospective protocol (PROSPERO CRD42017054445) [10] to perform this review according to the Preferred Reporting Items for Systematic Reviews and Meta-analyses (PRISMA) guidelines [11].

### Search strategy and selection criteria

#### Identification of relevant studies

We performed comprehensive electronic searches of major databases including PubMed/Medline and Embase using an African search filter developed by Eisinga and colleagues [12], to identify relevant studies published on CKD on the African continent between January 1, 2000 and December 31, 2016, without language restriction (Additional file 1: pp. 2–3). The searches were restricted to the post-2000 era in order to provide the most contemporary estimate of CKD prevalence in Africa. Estimates from previous studies are also likely based on older definitions of CKD and therefore not directly comparable to more recent studies. Additionally, we traced the citations of identified articles via the ISI Web of knowledge, and scanned the reference lists of review papers and conference proceedings. We also searched relevant African journals, the World Health Organization (WHO) Global Health Library databases (which include the African Index Medicus, WHO Library Information System, and Scientific Electronic Library Online).

#### Selection of included studies

Two investigators (ADK and TI) independently reviewed the articles by title, abstract, and full-text where relevant for inclusion. Disagreements were resolved by consensus or by consulting a third investigator (JBE). To be included in this review, primary studies had to be population or hospital- based, randomized controlled trial (RCT) or non-randomized studies including cross-sectional, cohort, and case-cohort studies that report prevalence or enough data to calculate prevalence of CKD in adults (aged 18 and above) from sub-Saharan and North African countries. For cohort studies or RCT, we included data from the baseline evaluation. CKD had to have been defined as the presence of kidney damage and/or estimated Glomerular filtration rate (eGFR) < 60 mL/min/1.73 m^2^, according to the NKF KDOQI (National Kidney Foundation Kidney disease outcomes quality initiative or KDIGO (Kidney Disease: Improving Global Outcomes) guidelines [13, 14]. We excluded studies in participants selected based on the presence or absence of kidney disease, studies limited to pregnant women. For multiple surveys conducted in different countries or at different calendar years and reported within the same article, each survey was accounted for separately, when it was possible to disaggregate the data by country.

### Assessment of the methodological quality of included studies

Two reviewers (ADK & TI) independently assessed study quality, with disagreements being resolved by consensus or by consulting a third investigator (JBE). We used the ten-item rating checklist developed by Hoy et al. that assesses sampling, the sampling technique and size, outcome measurement, response rate, and statistical reporting [15]. Each item was assigned a score of 1 (yes) or 0 (no), and scores were summed across items to generate an overall quality score ranging from 0 to 10. Each study was rated as being of low, moderate, or high methodological quality depending on the number of questions answered as “yes (low risk of bias)”. Studies of high quality had scores higher than 8, moderate a score of 6–8, and low a score of 5 or lower [15].

### Data extraction

The same reviewers independently selected studies and extracted relevant data using a pre-conceived extraction form. The information extracted included 1) author details (names and year of publication); 2) study characteristics (country, design, setting, data source, sampling method, sample size, data collection period, response rate); 3) Participants’ characteristics (age, gender, hypertension status, diabetes status, HIV status, HAART treatment); and 4) CKD characteristics (CKD diagnostic criteria, eGFR equation, proteinuria assessment method, prevalence, number of participants tested and diagnosed with CKD overall and by subgroups of interest).

### Statistical analysis

For each study, the unadjusted prevalence of CKD and standard errors were calculated (number of cases/sample size) based on the information on crude numerators and denominators provided in individual studies. CKD stages 1–5 was defined as kidney damage (urinary dipstick abnormalities) or eGFR < 60 ml/min/1.73m^2^, while CKD stages 3–5 was defined as eGFR < 60 ml/min/1.73m^2^. When eGFR from multiple equations was reported, we preferred the Chronic Kidney Disease Epidemiology Collaboration (CKD-EPI) equation, then the Modification of Diet in Renal Disease (MDRD), and lastly the Cockcroft-Gault formula in the main analyses. We used the DerSimonian-Laird random-effects models to generate the pooled prevalence of CKD according to each diagnostic criterion. The random effects model was chosen in anticipation of substantial variations in CKD prevalence estimates across the included studies. To minimize the effect of studies with extremely small or extremely large prevalence on the overall estimate, we stabilized the variance of the study-specific prevalence estimates with the Freeman-Tukey single arcsine transformation before pooling the data [16]. We examined prevalence by disease-specific populations (general population, HIV, Hypertension, diabetes mellitus), region, method of kidney disease assessment, setting, date. We assessed inter-rater agreement for inclusion and quality assessment using Cohen’s kappa (κ) coefficient.

We assessed heterogeneity between studies using Cochran’s Q statistic, H and the I^2^ statistics [17, 18], which estimate the percentage of total variation across studies due to true between-study difference rather than chance, with I^2^ values of 25, 50 and 75% representing low, medium and high heterogeneity, respectively. We explored sources of heterogeneity through a restriction of analyses to subgroups defined by geographical area (central, eastern, northern, southern and western Africa), sample size, and year of publication. Comparisons between subgroups were performed using the Q-test based on the Analysis of the Variance (ANOVA). Publication bias was evaluated using funnel plots supplemented by formal statistical assessment using the Egger’s test [19]. All analyses were performed using Stata software (Stata Corp V.14, Texas, USA).

## Results

### The review process

**Fig. 1:**
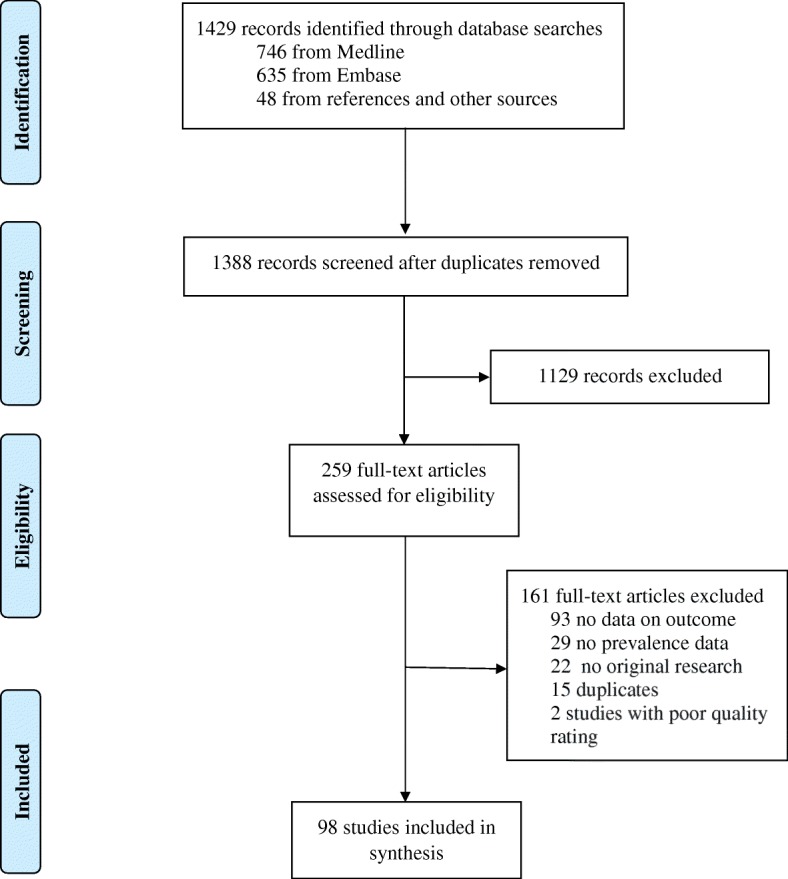
Selection of articles for inclusion in the systematic review

Fig. 1 summarizes the study selection process. In total, 1429 records were identified via databases searches. After removing duplicates, we scanned the titles and abstracts of 1388 studies, of which 259 were selected for full-text review. Of these, 98 met the inclusion criteria and were retained in the final review (Fig. 1). Inter-rater agreement for inclusion was excellent (κ = 0.965).

### Methodological quality of included studies

A total of 19 studies were deemed to be of high methodological quality, while 78 were categorized as being of moderate methodological quality; two studies were considered to be of low quality and were therefore excluded from the analysis (Additional file 1: Table S1, pp. 5–8). Inter-rater agreement for quality assessment was excellent (κ = 0.910).

### Characteristics of included studies

The characteristics of the included studies are summarized in Additional file 1: Table S2 (pp 9–13). Sixty four (65.3%) articles were published between 2012 and 2016. They originated from the five African sub-regions, with the western sub-region being the most represented (37 studies, 37.8%) and the northern being the least represented (six studies, 6.1%). Of the 54 African countries, 22 (40.7%) were represented in this systematic review including Nigeria (26 studies); South Africa, Ghana (nine studies each); Tanzania (eight studies); Cameroon, Democratic Republic of Congo (six studies each); Malawi (five studies); Uganda, Zimbabwe, Egypt, Kenya (three studies each); Congo, Sudan, Ethiopia, Senegal, Zambia (two studies each); Burundi, Morocco, Rwanda, Seychelles (one study each). One study presented combined data from two surveys conducted in Zimbabwe and Uganda, another study presented combined data from seven sub-Saharan countries (Cameroon, Ivory Coast Kenya, Mozambique, South Africa, Uganda, and Zambia). Of the included studies, 97 were local studies, mainly conducted in urban settings, while only one study had national coverage [20]. With regards to the study sites, the majority were solely hospital- or clinic-based (59 studies, 60.2%), with only 39 studies being community-based.

Data from 98,432 participants were included, with a median age of 43 years (25th–75th percentiles: 36.6 to 51.4). Fifty four (55.1%) studies examined participants thought be at high risk of CKD (people with hypertension, diabetes, HIV, or sickle cell disease), while 33 (33.7%) were conducted in the general population or subjects not known to be at risk of CKD, and 11 (11.2%) studies in both. The included studies applied various estimators of GFR. Fifty eight applied a single equation; the most frequently used being the Modification of Diet in Renal Disease (MDRD) eq. (32 studies), followed by the Cockcroft-Gault formula (17 studies), the Chronic Kidney Disease Epidemiology Collaboration (CKD-EPI) formula (7 studies), and the Cystatin C equation (two studies). Of the 22 studies that compared eGFR using two or more equations, Cockcroft-Gault and MDRD equations were used in 11 studies, CKD-EPI and MDRD used in 3 studies, and the three formulas (Cockcroft-Gault, CKD-EPI, and MDRD) used in 8 studies. The most common methods used to assess proteinuria were the urine dipstick test (46 studies), followed by the spot urine albumin to creatinine ratio (20 studies), and the 24-h urine collection (three studies).

### Prevalence of CKD in the general population

**Fig. 2:**
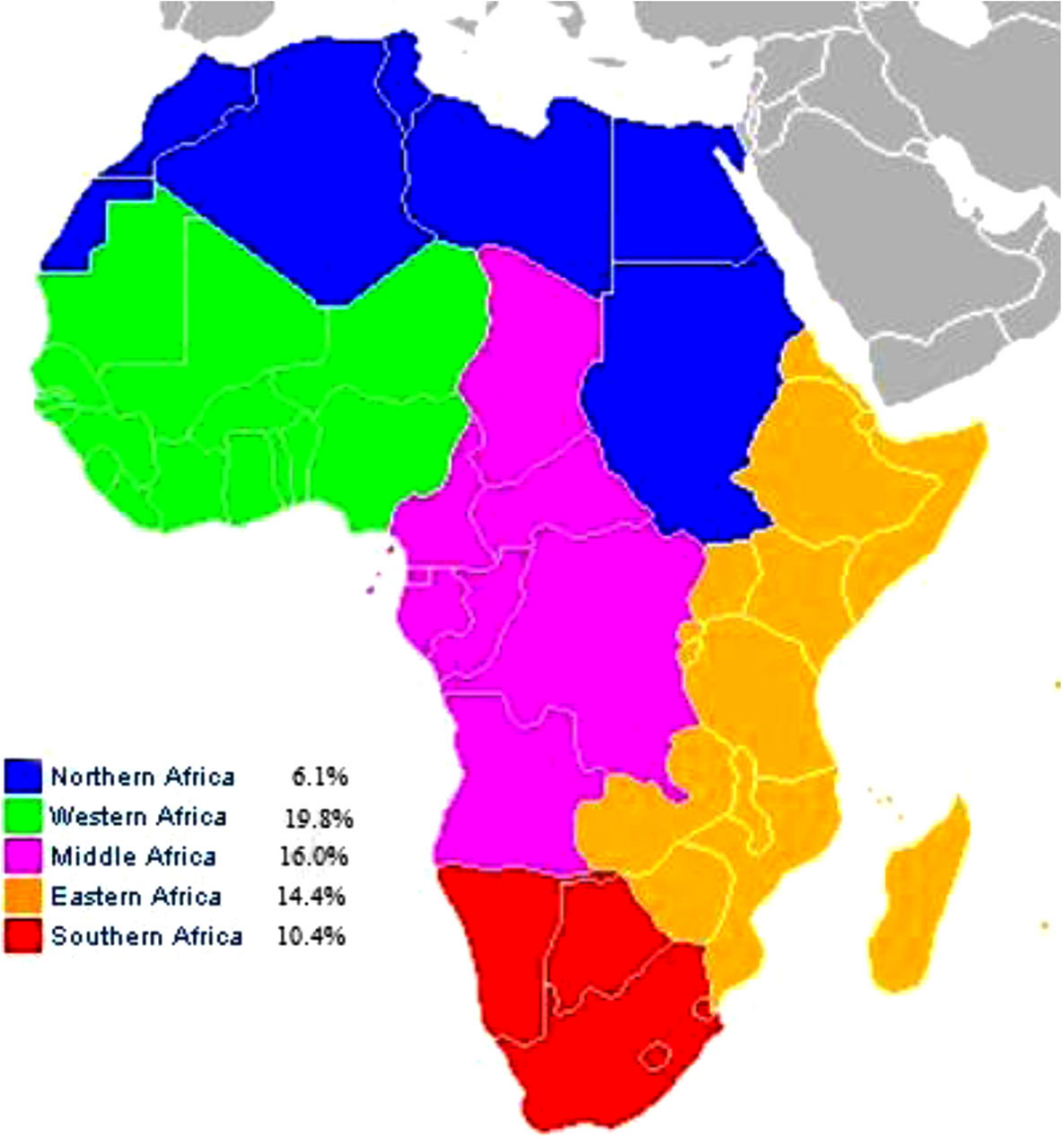
Prevalence of CKD in general populations of adults living on the African continent by region

**Fig. 3:**
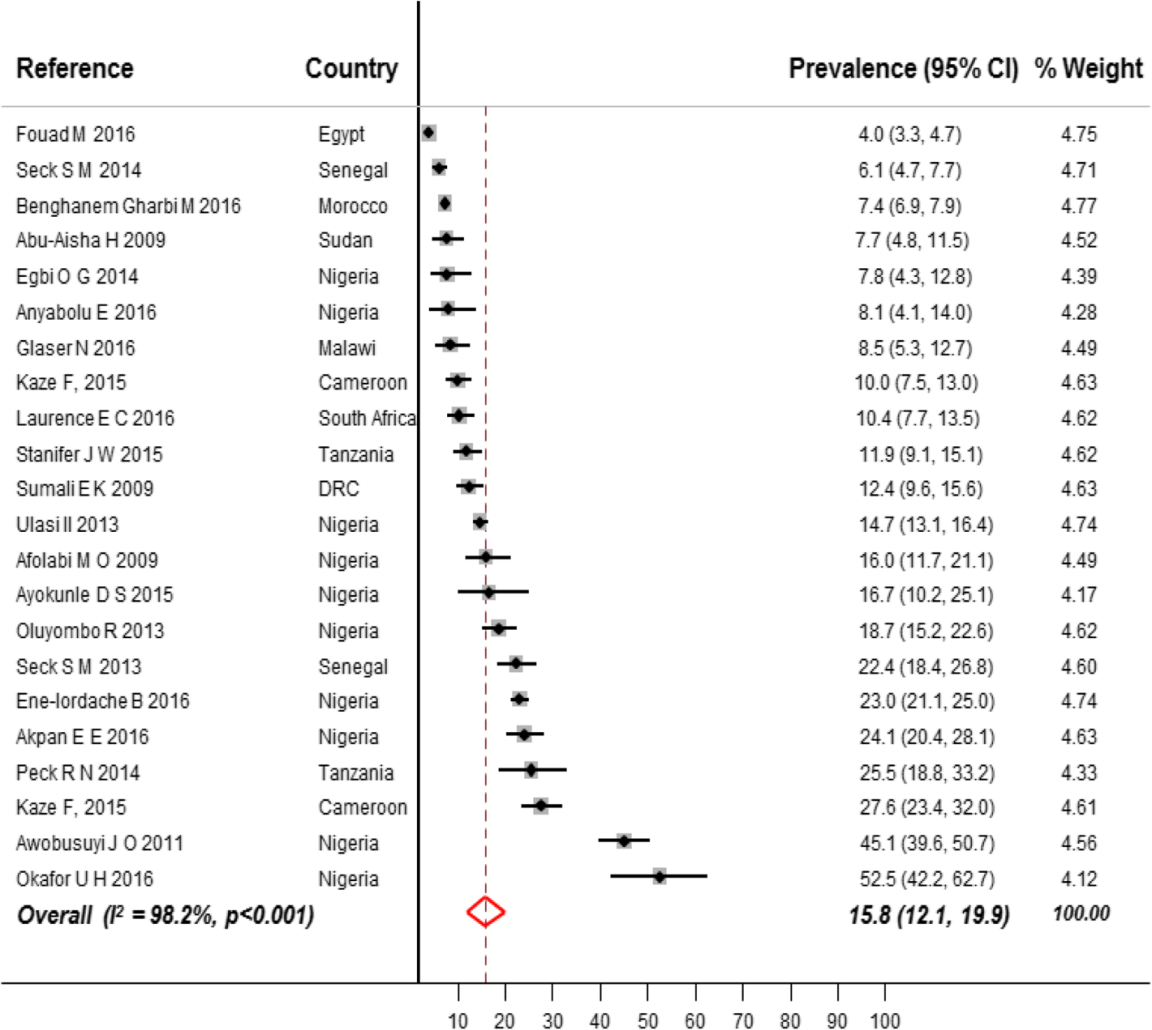
Prevalence of CKD in general populations of adults living in Africa. Black boxes represent the effect estimates (prevalence) and the horizontal bars are for the 95% confidence intervals (CIs). The diamond is for the pooled effect estimate and 95% CI and the dotted vertical line centered on the diamond has been added to assist visual interpretation

**Fig. 4:**
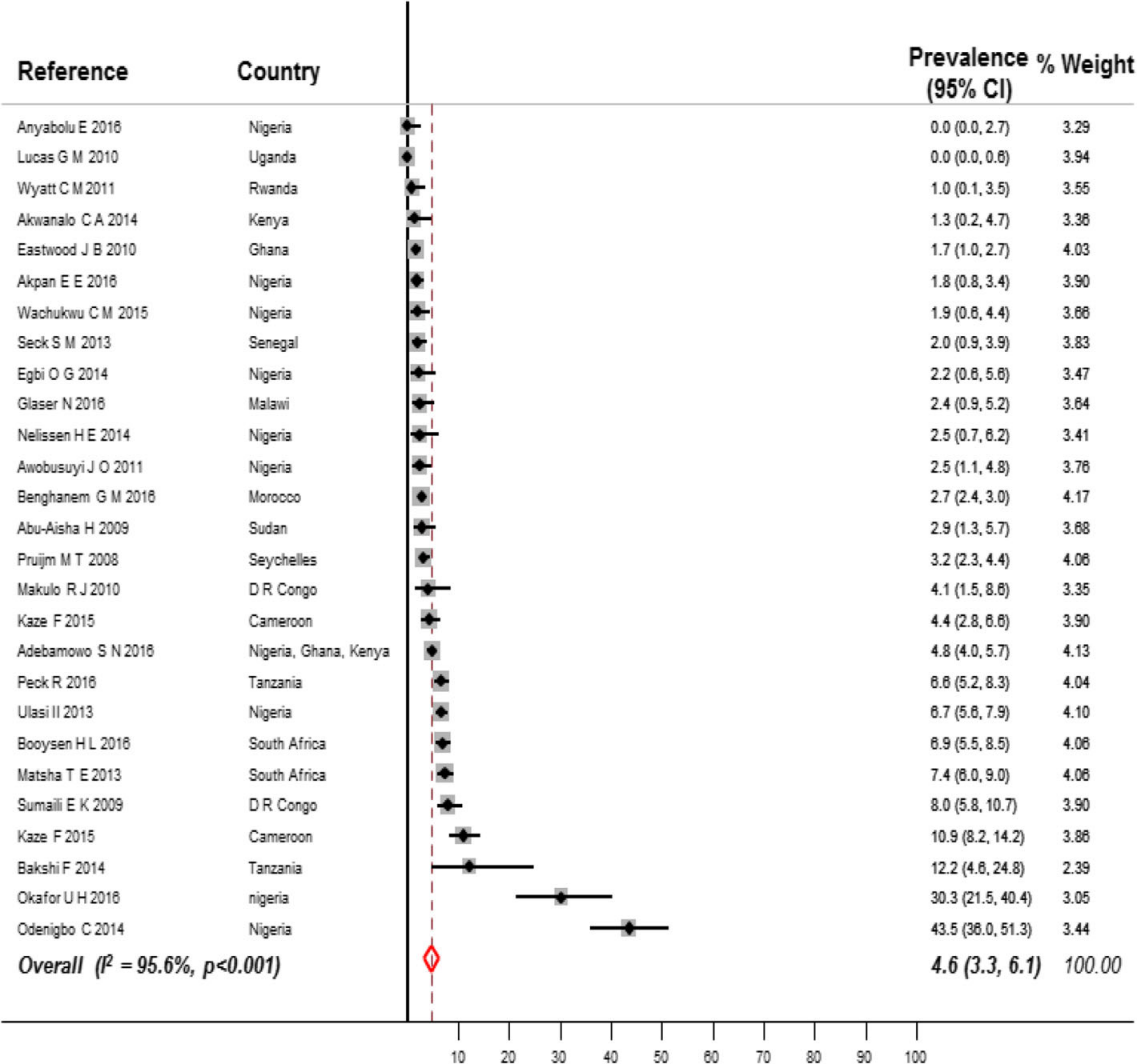
Prevalence of CKD stages 3 to 5 in general populations of adults living in Africa. Black boxes represent the effect estimates (prevalence) and the horizontal bars are for the 95% confidence intervals (CIs). The diamond is for the pooled effect estimate and 95% CI and the dotted vertical line centered on the diamond has been added to assist visual interpretation

Fig. 2 shows the prevalence of CKD in the general population by region. The overall prevalence in the general population was 15.8% (95% CI 12.1–19.9, *n* = 23,825, 22 studies, Fig. 3) for CKD stages 1 to 5, and 4.6% (95% CI 3.3–6.1, *n* = 25,929, 27 studies, Fig. 4) for CKD stages 3 to 5. The overall prevalence in the general population was significantly higher in sub-Saharan Africa compared to north Africa for both of CKD stages 1–5 (17.7, 95%CI 13.7–22.1; 10,028 participants vs 6.1, 95% CI 3.6–9.3, 13,797 participants, *p* < 0.001, Additional file 1: Table S3, pp. 14–15) and CKD stages 3–5 (4.8, 95% CI 3.2–6.6, *n* = 15,132 vs 2.6, 95% CI 2.3–2.9, *n* = 10,797; *p* = 0.004, Additional file 1: Table S4, pp. 15–16).

### Prevalence of CKD in high-risk populations

**Fig. 5:**
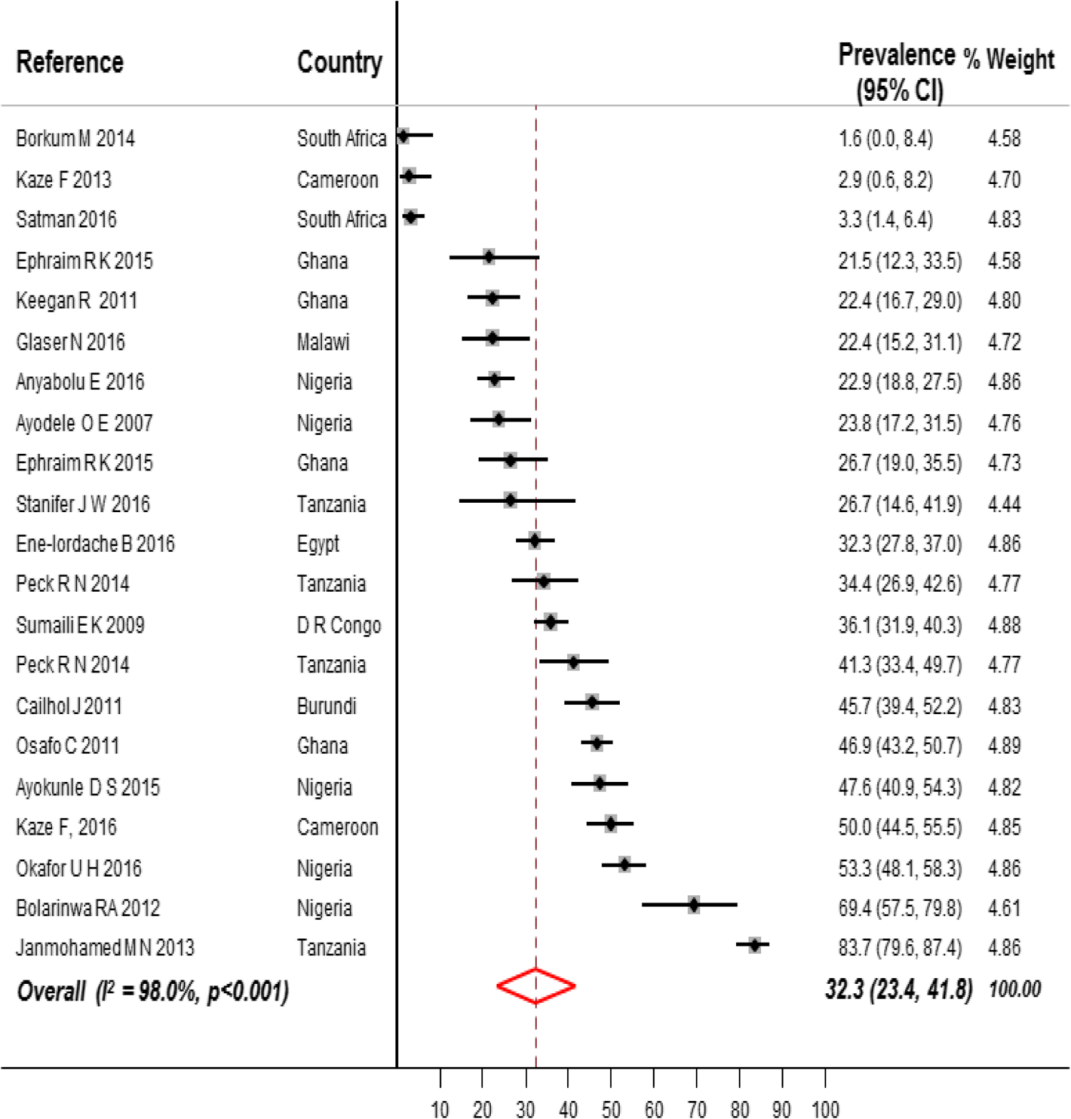
Prevalence of CKD in high-risk populations of adults living in Africa. Black boxes represent the effect estimates (prevalence) and the horizontal bars are for the 95% confidence intervals (CIs). The diamond is for the pooled effect estimate and 95% CI and the dotted vertical line centered on the diamond has been added to assist visual interpretation

**Fig. 6:**
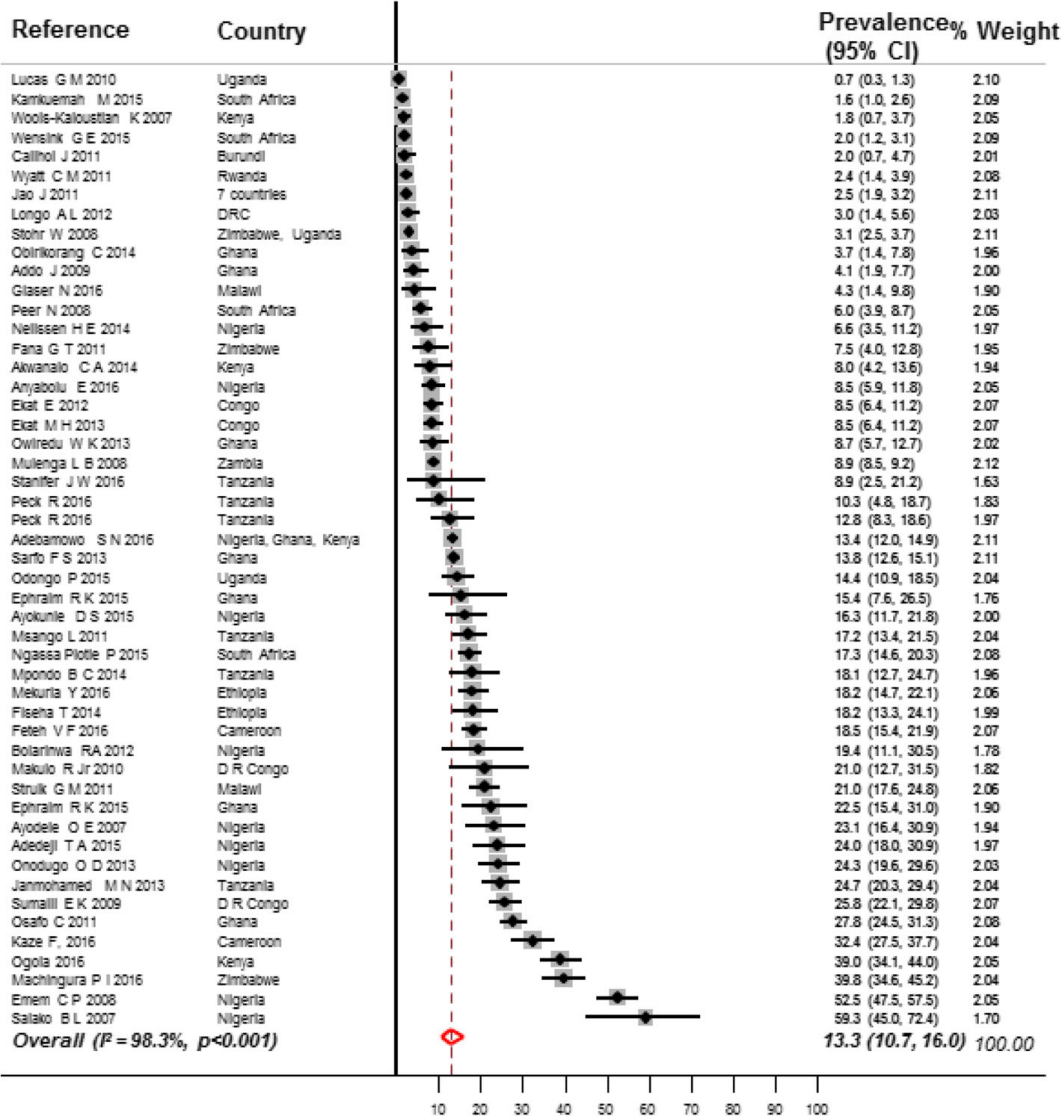
Prevalence of CKD stages 3 to 5 in high-risk populations of adults living in Africa. Black boxes represent the effect estimates (prevalence) and the horizontal bars are for the 95% confidence intervals (CIs). The diamond is for the pooled effect estimate and 95% CI and the dotted vertical line centered on the diamond has been added to assist visual interpretation

The prevalence of CKD stages 1 to 5 in the high-risk populations was 32.3% (95% CI 23.4–41.8, *n* = 5056; 21 contributions, Fig. 5) overall, 27.3% in participants with HIV (95% CI 17.0–38.9, *n* = 2007; 10 contributions, Additional file 1: Figure S3, pp. 23), 35.6% in participants with hypertension (95% CI 27.9–43.7, *n* = 2199, 6 contributions, Additional file 1: Figure S4, pp. 24), and 32.6% (95% CI 0.3–82.3, 778 participants, four contributions, Additional file 1: Figure S5, pp. 25) in participants with diabetes (Additional file 1: Table S5, pp. 16–17). The prevalence of CKD stages 3 to 5 in the high-risk populations was 13.3% (95% CI 10.7–16.0, 52,353 participants; 50 contributions, Fig. 6) overall, 9.1% (95% CI 6.6–11.9, *n* = 44,239, 28 contributions, Additional file 1: Figure S6, pp. 26) in participants with HIV, 17.9% (95% CI 10.9–26.1, *n* = 2971, 11 contributions, Additional file 1: Figure S7, pp. 27) in participants with hypertension, and 22.0% (95% CI 16.1–28.6, *n* = 5071, 10 contributions, Additional file 1: Figure S8, pp. 28) in participants with diabetes (Additional file 1: Table S6, pp. 17–18).

### Prevalence of CKD by eGFR estimating equation

In general populations, the prevalence of CKD stages 1–5 was 13.7% (95% CI 10.2–17.6, *n* = 23,825, 14 contributions) according to the MDRD equation, 21.3% (95% CI 9.9–35.5 *n* = 5316, 8 contributions) according to the Cockcroft-Gault formula, and 19.5% (95% CI 13.8–25.9, *n* = 4854, 5 contributions) when using the CKD-EPI formula (Additional file 1: Figure S1, pp. 19–20). The prevalence of CKD stages 3–5 was 4.5% (95% CI 3.0–6.3, *n* = 22,969, 21 contributions) by the MDRD equation, 11.8% (95% CI 7.3–17.1, *n* = 4927, 11 contributions) according to the Cockcroft-Gault formula, and 5.1% (95% CI 3.1–7.6, *n* = 6184, 7 contributions) by the CKD-EPI formula (Additional file 1: Figure S2, pp. 21–22).

In high-risk populations, the prevalence of CKD stages 1 to 5 was 27.7% (95% CI 17.1–39.7, *n* = 3262, 11 contributions) according to the MDRD equation, 49.8% (95% CI 28.8–70.8, *n* = 1361, six contributions) according to the Cockcroft-Gault formula, and 34.7% (95% CI 26.8–43.1, *n* = 1234, six contributions) based on the CKD-EPI formula (Additional file 1: Table S3, pp. 14). The prevalence of CKD stages 3 to 5 in high-risk populations was 10.6% (95% 7.6–13.9, *n* = 21,642, 31 contributions) according to the MDRD equation, 16.2% (95% CI 11.2–21.9, *n* = 40,610, 22 contributions) according to the Cockcroft-Gault formula, and 11.6% (95% CI 6.2–18.3, *n* = 6295, 11 contributions) based on the CKD-EPI formula (Additional file 1: Table S4, pp. 15–16).

The prevalence of proteinuria alone was 9.8% (95% CI 4.9–16.3, *n* = 8410; 11 studies) and 22.7% (95% CI 15.5–30.8, *n* = 8784; 26 studies) in the general and high-risk populations, respectively.

### Influence of year of publication, sample size, and median age

The prevalence of CKD stages 3–5 was significantly higher in studies reported after compared to before 2013 (5.7, 95% CI 4.0–7.8 vs 2.4, 95% CI 0.9–4.4, *p* = 0.02, Additional file 1: Table S4, pp. 15–16). Likewise and as expected, the prevalence of CKD stages 3–5 was significantly higher in older (≥ median age 43.7 years) compared to younger participants (*p* = 0.01, Additional file 1: Table S4, pp. 15–16). However, no difference in CKD prevalence was seen between studies larger and smaller than the median sample size (*p* = 0.08, Additional file 1: Table S3, pp. 14), studies with older compared to younger participants (*p* = 0.8, Additional file 1: Table S3, pp. 14), or studies reported before compared to after the median year of publication (*p* = 0.33, Additional file 1: Table S3, pp. 14).

### Investigation of the sources of heterogeneity and publication bias

We found substantial heterogeneity across the contributing studies overall, within subgroups for residence, median sample size, geographic region, and across eGFR estimating equations. There was no evidence of publication bias across studies reporting on the prevalence of CKD stages 1 to 5 in the general population, with the Egger test for bias yielding a *p*-value of 0.27 (Additional file 1: Table S3, pp. 14). We found some evidence of publication bias across studies reporting on the prevalence of CKD stages 1 to 5 in high-risk populations, with the Egger test for bias giving a p-value of 0.01 (Additional file 1: Table S5, pp. 16–17). However, smaller studies (sample size < median of 192) were not more likely to report more extreme results compared with larger studies (p-value = 0.11, Additional file 1: Table S5, pp. 16–17).

## Discussion

Our review including 98,432 individuals found a prevalence of 15.8% for CKD stages 1–5 in the general population of adults living on the African continent. Additionally, we showed that 4.6% of adults living in Africa have moderate or severe decreases in kidney function (i.e. CKD stages 3 to 5). The prevalence of CKD was higher in sub-Saharan Africa than North Africa, and nearly two times higher in high-risk populations than in general populations. The three main equations used to estimate the kidney also yielded different results. The Cockcroft formula showed a prevalence that was higher than prevalence obtained using MDRD or CKD-EPI equations. We found substantial heterogeneity across the studies and in subgroup analyses, and no evidence of publication bias across studies reporting on CKD prevalence in general populations of Africa.

Our review is the first to comprehensively assess the prevalence of CKD in adults living on the African continent. A previous systematic review on CKD prevalence limited to sub-Saharan Africa included articles published between 1962 and 2011; amongst which 32 (35.6%) were published before 2000 [9]. The vast majority (65.3%) of studies included in our review were published between 2012 and 2016, a more contemporary period. Our findings supplement previous studies and reviews on CKD by providing an updated and comprehensive synthesis of data on the magnitude of CKD in the African continent.

The CKD prevalence in this review is slightly higher than that reported in African countries in a recently published systematic review on the global CKD prevalence [21]. However, their review included data from three African countries (5497 individuals) and is therefore much less representative of the entire continent. The overall estimated prevalence of CKD stages 1–5 in the general population found in our review is similar to that (13.1%) found in the United States [22]. However, the prevalence of CKD in our review was higher than that found in general populations of adults living in four Asian countries (West Malaysia, Korea, China, and Taiwan) and Europe [23–27]. The prevalence of CKD stages 3–5 in our study was lower than that found in the United States (8.0%), and higher than that found in two Asian countries [23–27]. The prevalence of CKD in high-risk populations found in our review is similar to that found in a high-risk population (with diabetes or hypertension) in Korea (39.6%) [28]. Likewise, our estimate in high-risk subjects is comparable to the prevalence of CKD in a sample of hypertensive subjects in the Unites States [29].

While the attention of policy makers is finally extending beyond communicable diseases to the non-communicable diseases, particularly the cardiovascular disease (CVD) epidemic, it is not fully appreciated that this is accompanied by an epidemic of CKD. Our estimates indicate that CKD may be more common than diabetes which has an estimated prevalence of 3.2% in people aged 20 to 79 in sub-Saharan Africa [30]. The rapid rise in the number of people with hypertension or diabetes [7, 8], combined with the HIV pandemics, and the increased survival in individuals taking antiretroviral therapy are predicted to drive the burden of CKD in Africa [31]. Like hypertension or diabetes, CKD has consistently been shown to be associated with higher risk of mortality from CVD [26, 32, 33]. This situation is further compounded by the fact that a vast majority of people with CKD are unaware of their condition until they progress to later stages [34, 35]. Various observational cohort studies have shown that the increased risk in CVD mortality in CKD patients is apparent in the early stages of the disease, and nearly 40% of deaths from CKD occur prematurely (before age 65) [26, 32]. This highlights the need for interventions earlier in the process. Effective strategies can slow the progression of CKD and may help reduce the risk of CVD [14].

Our review points out the critical need of data in many parts of Africa that would help to further characterize the magnitude of CKD burden on the mother continent. Indeed, out of the 54 African countries, 32 were not included in this review. Although, the number of population-based studies on CKD prevalence has somewhat increased in the recent years, many African countries are still lagging behind. African countries must be encouraged to conduct population-based surveys of CKD prevalence such as the MAREMAR (Maladies Rénales Chroniques au Maroc) [36] project on a regular basis, in order to monitor time trends of CKD prevalence with comparable methodologies. African countries are also encouraged to incorporate CKD surveillance in existing data collection opportunities such as the WHO STEPwise approach to Surveillance surveys. Moreover, there is an urgent need for African nations to establish and sustain renal registries at both national and regional levels [37]. On a continent where access to healthcare is restricted due to economic constraints, the publication of registry data would be a cost-effective approach to draw the public and policy makers’ attention to the underappreciated problem of CKD, and help efforts to prevent, detect, and treat CKD at much earlier stages [37–39]. An African renal registry would facilitate the sharing of expertise across all the nations using this common platform, and lead to more effective patient advocacy, public health policy and fundraising [37].

Our review has some limitations. First, we found substantial heterogeneity in prevalence estimates, which was not completely explained by subgroup analyses. This may in part be explained by between-study differences in methodology and population structures, but they may also represent true regional differences in disease burden. Second, our ability to assess the quality of included studies was limited by the incomplete methodological information provided in some studies. Third, primary studies lacked data on important covariates that could have been used in meta-regression analyses to further explore and adjust for the sources of variations in prevalence between studies. Additionally, the majority of surveys did not follow patients for 3 months to confirm the diagnosis of CKD. Previous evidence suggested that a single measurement of eGFR may overestimate CKD prevalence [40]. These limitations notwithstanding, our study has several strengths. First, we used a comprehensive review protocol [10], and made extensive efforts to identify all the available evidence by searching multiple electronic databases without language restrictions; we applied an Africa-specific search filter [12], and adhered to pre-specified study selection criteria [10]. Second, we critically appraised the methodological quality of studies with a standard quality assessment tool for prevalence studies [15]. Finally, we used the Freeman-Tukey single arcsine transformation to stabilize the variance of prevalence estimates before pooling, therefore limiting the effects of studies with small and large prevalence estimates on the pooled estimates [16].

Key unaddressed issues in the detection of CKD in Africa include the absence of reliable and valid methods for assessing kidney function [41]. Our findings showed that CKD prevalence estimates can vary substantially depending on the equation used. Although the MDRD and CKD-EPI have been shown to be superior to the Cockcroft-Gault formula, the validity of those methods in African populations remains to be established [41, 42]. Furthermore, although it is widely accepted that CKD is associated with an increased all-cause and CVD mortality risks, the extent to which this applies to populations living on the African continent remains unclear. Most observational studies of adverse health outcomes in CKD patients were conducted in developed countries, which may not necessarily generalize to African populations [43]. African countries are encouraged to establish prospective multicenter cohorts of CKD patients such as the CRIC (Chronic Renal Insufficiency Cohort) Study [44], in order to examine risk factors for CKD progression and CVD tailored to their region, identify high-risk subgroups, and assess the role of genetic factors such as Apolipoprotein L1 (ApoL1) [45] variants in the genesis and progression of CKD in people living on the African continent.

## Conclusion

In summary, the substantial burden of CKD on the African continent found in this review highlights the need for a concerted action to prevent the high health and economic burden that this condition entails.

## Additional file

Additional file 1: Item 1: Search strategies. Item 2: Quality appraisal of included studies. Item 3: Supplementary tables. **Table S1.** Summary of the risk of bias in the included studies. **Table S2.** Characteristics of the 98 studies included in this systematic review. **Table S3.** Summary statistics for prevalence of CKD stages 1–5 in general populations. **Table S4.** Summary statistics for prevalence of CKD stages 3–5 in general populations. **Table S5.** Summary statistics for prevalence of CKD stages 1–5 in high-risk populations. **Table S6.** Summary statistics for prevalence of CKD stages 3–5 in high-risk populations. Item 4: Supplementary figures. **Figure S1.** Prevalence of CKD in the general population of Africa according to eGFR equation. **Figure S2.** Prevalence of CKD stages 3 to 5 in the general population of Africa according to eGFR equation. **Figure S3.** Prevalence of CKD in HIV-positive individuals living in Africa. **Figure S4.** Prevalence of CKD in hypertensive individuals living in Africa. **Figure S5.** Prevalence of CKD in people with diabetes mellitus living in Africa. **Figure S6.** Prevalence of CKD stages 3 to 5 in HIV-positive individuals living in Africa. **Figure S7.** Prevalence of CKD stages 3 to 5 in hypertensive individuals living in Africa. **Figure S8.** Prevalence of CKD stages 3 to 5 in people with diabetes mellitus living in Africa. (PDF 1107 kb).

## Abbreviations

ANOVA: Analysis of the Variance
ApoL1: Apolipoprotein L1
CKD: Chronic kidney disease
CKD-EPI: Chronic kidney disease epidemiology collaboration
CRIC: Chronic Renal Insufficiency Cohort
CVD: Cardiovascular disease
eGFR: Estimated glomerular filtration rate
ESRD: End-stage renal disease
HAART: Highly active antiretroviral therapy
HIV: Human immunodeficiency virus
KDIGO: Kidney disease: improving global outcomes
MAREMAR: Maladies Rénales Chroniques au Maroc
MDRD: Modification of Diet in Renal Disease
NKF KDOQI: National kidney foundation Kidney disease outcomes quality initiative
PRISMA: Preferred Reporting Items for Systematic Reviews and Meta-analyses
RCT: Randomized controlled trial
RRT: Renal replacement therapy
WHO: World health organization
