# Supplementary material for: Burden of chronic kidney disease on the African continent: a systematic review and meta-analysis
Source: BMC Nephrol. 2018 Jun 1;19:125. doi: 10.1186/s12882-018-0930-5 (PMC5984759; doi:10.1186/s12882-018-0930-5)
Supplement: Supplementary file 1 — Item 1: Search strategies. Item 2: Quality appraisal of included studies. Item 3: Supplementary tables. Table S1. Summary of the risk of bias in the included studies. Table S2. Characteristics of the 98 studies included in this systematic review. Table S3. Summary statistics for prevalence of CKD stages 1–5 in general populations. Table S4. Summary statistics for prevalence of CKD stages 3–5 in general populations. Table S5. Summary statistics for prevalence of CKD stages 1–5 in high-risk populations. Table S6. Summary statistics for prevalence of CKD stages 3–5 in high-risk populations. Item 4: Supplementary figures. Figure S1. Prevalence of CKD in the general population of Africa according to eGFR equation. Figure S2. Prevalence of CKD stages 3 to 5 in the general population of Africa according to eGFR equation. Figure S3. Prevalence of CKD in HIV-positive individuals living in Africa. Figure S4. Prevalence of CKD in hypertensive individuals living in Africa. Figure S5. Prevalence of CKD in people with diabetes mellitus living in Africa. Figure S6. Prevalence of CKD stages 3 to 5 in HIV-positive individuals living in Africa. Figure S7. Prevalence of CKD stages 3 to 5 in hypertensive individuals living in Africa. Figure S8. Prevalence of CKD stages 3 to 5 in people with diabetes mellitus living in Africa. (PDF 1107 kb). [file 12882_2018_930_MOESM1_ESM.pdf]

SUPPLEMENTARY MATERIAL

Item 1: Search strategies

Item 2: Quality appraisal of included studies

Item 3: Supplementary tables

- Table S1: Summary of the risk of bias in the included studies
- Table S2: Characteristics of the 98 studies included in this systematic review
- Table S3: Summary statistics for prevalence of CKD stages 1-5 in general populations
- Table S4: Summary statistics for prevalence of CKD stages 3-5 in general populations
- Table S5: Summary statistics for prevalence of CKD stages 1-5 in high-risk populations
- Table S6: Summary statistics for prevalence of CKD stages 3-5 in high-risk populations

Item 4: Supplementary figures

- Figure S1: Prevalence of CKD in the general population of Africa according to eGFR equation
- Figure S2: Prevalence of CKD stages 3 to 5 in the general population of Africa according to eGFR equation
- Figure S3: Prevalence of CKD in HIV-positive individuals living in Africa
- Figure S4: Prevalence of CKD in hypertensive individuals living in Africa
- Figure S5: Prevalence of CKD in people with diabetes mellitus living in Africa
- Figure S6: Prevalence of CKD stages 3 to 5 in HIV-positive individuals living in Africa
- Figure S7: Prevalence of CKD stages 3 to 5 in hypertensive individuals living in Africa
- Figure S8: Prevalence of CKD stages 3 to 5 in people with diabetes mellitus living in Africa

Item 1: Search strategies

The African Search Filter

(‘‘Africa’’[MeSH] OR Africa*[tw] OR Algeria[tw] OR Angola[tw] OR Benin[tw] OR Botswana[tw] OR ‘‘Burkina Faso’’[tw] OR Burundi[tw] OR Cameroon[tw] OR ‘‘Canary Islands’’[tw] OR ‘‘Cape Verde’’[tw] OR ‘‘Central African Republic’’[tw] OR Chad[tw] OR Comoros[tw] OR Congo[tw] OR ‘‘Democratic Republic of Congo’’[tw] OR Djibouti[tw] OR Egypt[tw] OR ‘‘Equatorial Guinea’’[tw] OR Eritrea[tw] OR Ethiopia[tw] OR Gabon[tw] OR Gambia[tw] OR Ghana[tw] OR Guinea[tw] OR ‘‘Guinea Bissau’’[tw] OR ‘‘Ivory Coast’’[tw] OR ‘‘Cote d’Ivoire’’[tw] OR Jamahiriya[tw] OR Jamahiriya[tw] OR Kenya[tw] OR Lesotho[tw] OR Liberia[tw] OR Libya[tw] OR Libya[tw] OR Madagascar[tw] OR Malawi[tw] OR Mali[tw] OR Mauritania[tw] OR Mauritius[tw] OR Mayotte[tw] OR Morocco[tw] OR Mozambique[tw] OR Mozambique[tw] OR Namibia[tw] OR Niger[tw] OR Nigeria[tw] OR Principe[tw] OR Reunion[tw] OR Rwanda[tw] OR ‘‘Sao Tome’’[tw] OR Senegal[tw] OR Seychelles[tw] OR ‘‘Sierra Leone’’[tw] OR Somalia[tw] OR ‘‘South Africa’’[tw] OR ‘‘St Helena’’[tw] OR Sudan[tw] OR Swaziland[tw] OR Tanzania[tw] OR Togo[tw] OR Tunisia[tw] OR Uganda[tw] OR ‘‘Western Sahara’’[tw] OR Zaire[tw] OR Zambia[tw] OR Zimbabwe[tw] OR ‘‘Central Africa’’[tw] OR ‘‘Central African’’[tw] OR ‘‘West Africa’’[tw] OR ‘‘West African’’[tw] OR ‘‘Western Africa’’[tw] OR ‘‘Western African’’[tw] OR ‘‘East Africa’’[tw] OR ‘‘East African’’[tw] OR ‘‘Eastern Africa’’[tw] OR ‘‘Eastern African’’[tw] OR ‘‘North Africa’’[tw] OR ‘‘North African’’[tw] OR ‘‘Northern Africa’’[tw] OR ‘‘Northern African’’[tw] OR ‘‘South African’’[tw] OR ‘‘Southern Africa’’[tw] OR ‘‘Southern African’’[tw] OR ‘‘sub Saharan Africa’’[tw] OR ‘‘sub Saharan African’’[tw] OR ‘‘sub-Saharan Africa’’[tw] OR ‘‘sub-Saharan African’’[tw]) NOT (‘‘guinea pig’’[tw] OR ‘‘guinea pigs’’[tw] OR ‘aspergillums Niger’’[tw])

PubMed search strategy for systematic review of burden of chronic kidney disease on the African continent

("Prevalence"[Mesh] OR "Epidemiology"[Mesh] OR Prevalence[tiab] OR epidemiology[tiab])

AND ("Kidney Diseases"[Mesh] OR "Renal Insufficiency, Chronic"[Mesh] OR "Proteinuria"[Mesh] OR "Albuminuria"[Mesh] OR Chronic Kidney Disease[tiab] OR Kidney Disease[tiab] OR Nephropathy[tiab] OR Renal Insufficiency[tiab] OR Renal Impairment[tiab] OR Impaired Kidney Function[tiab] OR ckd[tiab] OR crd[tiab] OR eskd[tiab] OR End Stage Renal Disease[tiab] OR Kidney Disease[tiab] OR Dialysis[tiab] OR Chronic Renal Failure[tiab] OR Renal Failure[tiab] OR Proteinuria[tiab] OR albuminuria[tiab])

AND ("Africa"[Mesh] OR Africa[tiab] OR "Africa South of the Sahara"[Mesh] OR "Africa, Western"[Mesh] OR "Africa, Southern"[Mesh] OR "Africa, Northern"[Mesh] OR "Africa, Eastern"[Mesh] OR "Africa, Central"[Mesh] OR Angola[tiab] OR Benin[tiab] OR Botswana[tiab] OR Burkina Faso[tiab] OR Upper Volta[tiab] OR Burundi[tiab] OR Urundi[tiab] OR Cameroon[tiab] OR Cameroons[tiab] OR Cape Verde[tiab] OR Central African Republic[tiab] OR Chad[tiab] OR Comoros[tiab] OR Comoro Islands[tiab] OR Comores[tiab] OR Mayotte[tiab] OR Congo[tiab] OR Zaire[tiab] OR Cote d'Ivoire[tiab] OR Ivory Coast[tiab] OR Democratic Republic of the Congo[tiab] OR Djibouti[tiab] OR French Somaliland[tiab] OR Eritrea[tiab] OR Ethiopia[tiab] OR Gabon[tiab] OR Gabonese Republic[tiab] OR Gambia[tiab] OR Ghana[tiab] OR Gold Coast[tiab] OR Guinea[tiab] OR Kenya[tiab] OR Lesotho[tiab] OR Basutoland OR Liberia[tiab] OR Madagascar[tiab] OR Malagasy Republic[tiab] OR Malawi[tiab] OR Nyasaland[tiab] OR Mali[tiab] OR Mauritania[tiab] OR Mauritius[tiab] OR Mozambique[tiab] OR Namibia[tiab] OR Niger OR Nigeria[tiab] OR Rwanda[tiab] OR Sao Tome[tiab] OR Seychelles[tiab] OR Senegal[tiab] OR Sierra Leone[tiab] OR Somalia[tiab] OR South Africa[tiab] OR Sudan[tiab] OR Swaziland[tiab] OR Tanzania[tiab] OR Togo[tiab] OR Togolese Republic[tiab] OR Uganda[tiab] OR Zambia[tiab] OR Zimbabwe[tiab] OR Rhodesia[tiab] OR Algeria[tiab] OR Egypt[tiab] OR Lybia[tiab] OR Morocco[tiab] OR Tunisia[tiab] OR Western Sahara[tiab])

Filters: Publication date from 2000/01/01 to 2016/12/31; Humans

Embase search strategy for systematic review of burden of chronic kidney disease on the African continent

'prevalence'/exp OR 'epidemiology'/exp OR prevalence:ab,ti OR epidemiology:ab,ti

AND ('chronic kidney disease'/exp OR 'chronic kidney failure'/exp OR 'end stage renal disease'/exp OR 'proteinuria'/exp OR 'chronic kidney disease':ab,ti OR 'nephropathy':ab,ti OR 'renal insufficiency':ab,ti OR 'renal impairment':ab,ti OR 'impaired kidney function':ab,ti OR ckd:ab,ti OR crd:ab,ti OR eskd:ab,ti OR 'end stage renal disease':ab,ti OR 'kidney disease':ab,ti OR dialysis:ab,ti OR 'chronic renal failure':ab,ti OR 'renal failure':ab,ti OR proteinuria:ab,ti OR albuminuria:ab,ti)

AND ('africa'/exp OR 'north africa'/exp OR 'africa south of the sahara'/exp OR 'sub saharan africa':ab,ti OR 'subsaharan africa':ab,ti OR angola:ab,ti OR benin:ab,ti OR botswana:ab,ti OR 'burkina faso':ab,ti OR 'upper volta':ab,ti OR burundi:ab,ti OR urundi:ab,ti OR cameroon:ab,ti OR cameroons:ab,ti OR 'cape verde':ab,ti OR 'central african republic':ab,ti OR chad:ab,ti OR comoros:ab,ti OR 'comoro islands':ab,ti OR comores:ab,ti OR mayotte:ab,ti OR 'congo'/exp OR 'ivory coast':ab,ti OR 'democratic republic of the congo':ab,ti OR djibouti:ab,ti OR 'french somaliland':ab,ti OR eritrea:ab,ti OR ethiopia:ab,ti OR gabon:ab,ti OR 'gabonese republic':ab,ti OR gambia:ab,ti OR ghana:ab,ti OR 'gold coast':ab,ti OR guinea:ab,ti OR kenya:ab,ti OR lesotho:ab,ti OR basutoland:ab,ti OR liberia:ab,ti OR madagascar:ab,ti OR 'malagasy republic':ab,ti OR malawi:ab,ti OR nyasaland:ab,ti OR mali:ab,ti OR mauritania:ab,ti OR mauritius:ab,ti OR mozambique:ab,ti OR namibia:ab,ti OR niger:ab,ti OR nigeria:ab,ti OR rwanda:ab,ti OR 'sao tome':ab,ti OR seychelles:ab,ti OR senegal:ab,ti OR 'sierra leone':ab,ti OR somalia:ab,ti OR 'south africa':ab,ti OR sudan:ab,ti OR swaziland:ab,ti OR tanzania:ab,ti OR togo:ab,ti OR 'togolese republic':ab,ti OR uganda:ab,ti OR zambia:ab,ti OR zimbabwe:ab,ti OR rhodesia:ab,ti OR algeria:ab,ti OR egypt:ab,ti OR lybia:ab,ti OR morocco:ab,ti OR tunisia:ab,ti OR 'western sahara':ab,ti)

Filters: Publication date from 2000/01/01 to 2016/12/31; Humans

Item 2: Quality appraisal of included studies

Quality assessment criteria for prevalence studies

Items	Quality score
**External validity**	
1. Was the study's target population a close representation of the national population in relation to relevant variables?	(1 point)
2. Was the sampling frame a true or close representation of the target population?	(1 point)
3. Was some form of random selection used to select the sample, OR was a census undertaken?	(1 point)
4. Was the likelihood of nonresponse bias minimal?	(1 point)
	Total (4 points)
**Internal validity**	
5. Were data collected directly from the subjects (as opposed to a proxy)?	(1 point)
6. Was an acceptable case definition used in the study?	(1 point)
7. Was the study instrument that measured the parameter of interest shown to have validity and reliability?	(1 point)
8. Was the same mode of data collection used for all subjects?	(1 point)
9. Was the length of the shortest prevalence period for the parameter of interest appropriate?	(1 point)
10. Were the numerator(s) and denominator(s) for the parameter of interest appropriate?	(1 point)
	Total (6 points)

Source: Hoy et al **[2]**.

Methodological quality:

High quality: score > 8

Moderate quality: score 6-8

Low quality: score 0-5

Item 3: Supplementary tables

Table S1: Summary of the risk of bias in the included studies

		External validity				Internal validity							
**Author Year**	**Country**	**Risk of bias 1**	**Risk of bias 2**	**Risk of bias 3**	**Risk of bias 4**	**Risk of bias5**	**Risk of bias 6**	**Risk of bias 7**	**Risk of bias 8**	**Risk of bias 9**	**Risk of bias 10**	**Score**	**Quality**
Adebamowo S N 2016	Nigeria, Ghana, Kenya	0	1	0	1	1	1	1	1	1	1	8	moderate
Adedeji T A 2015	Nigeria	0	1	0	NCS	1	1	1	1	1	1	7	moderate
Afolabi M O 2009	Nigeria	0	1	0	NCS	1	1	1	1	1	1	7	moderate
Akwanalo C A 2014	Kenya	0	1	0	1	1	1	1	1	1	1	8	moderate
Anyabolu E 2016	Nigeria	0	1	0	1	1	1	1	1	1	1	8	moderate
Awobusuyi J O 2011	Nigeria	0	1	0	1	1	1	1	1	1	1	8	moderate
Ayokunle D S 2015	Nigeria	0	1	0	1	1	1	1	1	1	1	8	moderate
Bakshi F 2014	Tanzania	0	1	0	NCS	1	1	1	1	1	1	7	moderate
Benghanem Gharbi M 2016	Morocco	0	1	1	1	1	1	1	1	1	1	9	high
Booysen H L 2016	South Africa	0	1	1	1	1	1	1	1	1	1	9	high
Borkum M 2014	South Africa	0	1	0	0	1	1	1	1	1	1	7	moderate
Brenyah R C 2013	Ghana	0	1	1	1	1	1	1	1	1	1	9	high
Cailhol J 2011	Burundi	0	1	0	1	1	1	1	1	1	1	8	moderate
Egbi O G 2014	Nigeria	0	1	0	NCS	1	1	1	1	1	1	7	moderate
Ekat M H 2013	Congo	0	1	0	0	1	1	1	1	1	1	7	moderate
El-Tayeb M 2010	Egypt	0	1	1	1	1	1	1	1	1	1	9	high
Emem C P 2008	Nigeria	0	1	0	NCS	1	1	1	1	1	1	7	moderate
Ene-Iordache B 2016	Nigeria	0	1	0	NCS	1	1	1	1	1	1	7	moderate
Ephraim R K 2015	Ghana	0	1	0	1	1	1	1	1	1	1	8	moderate
Fana G T 2011	Zimbabwe	0	1	0	NCS	1	1	1	1	1	1	7	moderate
Feteh V F 2016	Cameroon	0	1	0	1	1	1	1	1	1	1	8	moderate
Fiseha T 2014	Ethiopia	0	1	1	NCS	1	1	1	1	1	1	8	moderate
Glaser N 2013	Malawi	0	1	0	1	1	1	1	1	1	1	8	moderate
Janmohamed M N 2013	Tanzania	0	1	0	1	1	1	1	1	1	1	8	moderate

Jao J 2011	7 countries	0	1	0	NCS	1	1	1	1	1	1	7	moderate
Kamkuemah M 2015	South Africa	0	1	0	1	1	1	1	1	1	1	8	moderate
Kaze F 2013	Cameroon	0	1	0	NCS	1	1	1	1	1	1	7	moderate
Kaze F, 2015 (500)	Cameroon	0	1	1	1	1	1	1	1	1	1	9	high
Kaze F, 2015 (439)	Cameroon	0	1	1	1	1	1	1	1	1	1	9	high
Kaze F, 2016	Cameroon	0	1	0	1	1	1	1	1	1	1	8	moderate
Keegan R 2011	Ghana	0	1	0	NCS	1	1	1	1	1	1	7	moderate
Longo A L 2012	DR Congo	0	1	0	NCS	1	1	1	1	1	1	7	moderate
Lucas G M 2010	Uganda	0	1	1	1	1	1	1	1	1	1	9	high
Lunyera J 2016	Uganda	0	1	0	NCS	1	1	1	1	1	1	7	moderate
Mafundikwa A 2007	Zimbabwe	0	1	0	NCS	1	1	1	1	1	1	7	moderate
Makusidi M A 2013	Nigeria	0	1	1	1	1	1	1		1	1	8	moderate
Masimango M I 2014	DR Congo	0	1	0	NCS	1	1	1	1	1	1	7	moderate
Matsha T E 2013	South Africa	0	1	1	1	1	1	1	1	1	1	9	high
Motala A A 2001	South Africa	0	1	0	NCS	0	1	0	1	1	1	5	low
Msango L 2011	Tanzania	0	1	0	1	1	1	1	1	1	1	8	moderate
Mulenga L B 2008	Zambia	0	1	0	1	1	1	1	1	1	1	8	moderate
Nelissen H E 2014	Nigeria	0	1	1	NCS	1	1	1	1	1	1	7	moderate
Ngassa Piotie P 2015	South Africa	0	1	0	NCS	1	1	1	1	1	1	7	moderate
Obirikorang C 2014	Ghana	0	1	0	NCS	1	1	1	1	1	1	7	moderate
Odenigbo C 2014	Nigeria	0	1	0	1	1	1	1	1	1	1	8	moderate
Odongo P 2015	Uganda	0	1	0	1	1	1	1	1	1	1	8	moderate
Okafor U H 2016	Nigeria	0	1	0	NCS	1	1	1	1	1	1	7	moderate
Oladapo O O 2012	Nigeria	0	1	0	NCS	1	1	0	1	1	1	7	moderate
Olanrewaju T O 2010	Nigeria	0	1	0	0	1	1	1	1	1	1	7	moderate
Onodugo O D 2013	Nigeria	0	1	0	0	1	1	1	1	1	1	7	moderate
Osafo C 2011	Ghana	0	1	0	0	1	1	1	1	1	1	7	moderate
Peck R 2016	Tanzania	0	1	1	1	1	1	1	1	1	1	9	high

Peck R N 2014	Tanzania	0	1	0	1	1	1	1	1	1	1	8	moderate
Peer N 2008	South Africa	0	1	0	0	1	1	1	1	1	1	7	moderate
Pruijm M T 2008	Seychelles	1	1	1	1	1	1	1	1	1	1	10	high
Rasmussen J B 2016	Zambia	0	1	0	0	1	1	1	1	1	1	7	moderate
Salako B L 2007	Nigeria	0	1	0	0	1	1	1	1	1	1	7	moderate
Sarfo F S 2013	Ghana	0	1	0	1	1	1	1	1	1	1	8	moderate
Seck S M 2013	Senegal	0	1	1	1	1	1	1	1	1	1	9	high
Seck S M 2014	Senegal	0	1	1	1	1	1	1	1	1	1	9	high
Stanifer J W 2015	Tanzania	0	1	1	1	1	1	1	1	1	1	9	high
Struik G M 2011	Malawi	0	1	0	1	1	1	1	1	1	1	8	moderate
Sumaili E K 2009	DR Congo	0	1	1	1	1	1	1	1	1	1	9	high
Sumaili E K 2009	DR Congo	0	1	0	1	1	1	1	1	1	1	8	moderate
Wensink G E 2015	South Africa	0	1	1	NCS	1	1	1	1	1	1	8	moderate
Wools-Kaloustian K 2007	Kenya	0	1	0	NCS	1	1	1	1	1	1	7	moderate
Wyatt C M 2011	Rwanda	0	1	0	NCS	1	1	1	1	1	1	7	moderate
Wachukwu C M 2015	Nigeria	0	1	0	NCS	1	1	1	1	1	1	7	moderate
Oluyombo R 2013	Nigeria	0	1	1	NCS	1	1	1	1	1	1	7	moderate
Ekat E 2012	Congo	0	1	0	NCS	1	1	1	1	1	1	7	moderate
Makulo R Jr 2010	DR Congo	0	1	1	NCS	1	1	1	1	1	1	8	moderate
Ayodele O E 2007	Nigeria	0	1	0	NCS	1	1	1	1	1	1	7	moderate
Ayodele O E 2011	Nigeria	0	1	0	NCS	1	1	1	1	1	1	7	moderate
Machingura P I 2016	Zimbabwe	0	1	0	1	1	1	1	1	1	1	8	moderate
Akpan E E 2016	Nigeria	0	1	0	NCS	1	1	1	1	1	1	7	moderate
Divala O H 2016	Malawi	0	1	0	NCS	1	1	1	1	1	1	7	moderate
Glaser N 2016	Malawi	0	1	0	1	1	1	1	1	1	1	8	moderate
Laurence E C 2016	South Africa	0	1	1	NCS	1	1	1	1	1	1	8	moderate
Satman 2016	South Africa	0	1	1	NCS	1	1	1	1	1	1	8	moderate
Mekuria Y 2016	Ethiopia	0	1	0	NCS	1	1	1	1	1	1	7	moderate
Okafor U H 2016	Nigeria	0	1	0	1	1	1	1	1	1	1	8	moderate
Stanifer J W 2016	Tanzania	0	1	1	1	1	1	1	1	1	1	9	high

Fouad M 2016	Egypt	0	1	0	1	1	1	1	1	1	1	8	moderate
Ogola 2016	Kenya	0	1	0	NCS	1	1	1	1	1	1	7	moderate
Owiredu W K 2013	Ghana	0	1	0	NCS	1	1	1	1	1	1	7	moderate
Beheiry H 2015	Sudan	0	1	1	NCS	1	1	1	1	1	1	8	moderate
Bolarinwa RA 2012	Nigeria	0	1	0	NCS	1	1	1	1	1	1	7	moderate
Abdu 2011	Nigeria	0	1	0	NCS	1	1	1	1	1	1	7	moderate
Cohen D B 2010	Malawi	0	1	0	1	1	1	1	1	1	1	8	moderate
Ebong C E 2007	Cameroon	0	1	0	NCS	1	1	1	1	1	1	7	moderate
Mpondo B C 2014	Tanzania	0	1	0	1	1	1	1	1	1	1	8	moderate
Sumaili E K 2008	R Congo	0	1	0	1	1	1	1	1	1	1	8	moderate
Stohr W 2008	Zimbabwe, Uganda	0	1	1	1	1	1	1	1	1	1	9	high
Addo J 2009	Ghana	0	1	0	1	1	1	1	1	1	1	8	moderate
Eastwood J B 2010	Ghana	0	1	1	1	1	1	1	1	1	1	9	high
Abu-Aisha H 2009	Sudan	0	1	1	1	1	1	1	1	1	1	9	high
Fatiu A 2011	Nigeria	0	1	0	NCS	1	1	1	1	1	1	7	moderate
Ulasi II 2013	Nigeria	0	1	1	1	1	1	1	1	1	1	9	high
Wondifraw Baynes H 2016	Ethiopia	0	1	0	0	0	1	1	0	0	1	4	low

NCS: Not clearly stated

Table S2: Characteristics of the 98 studies included in this systematic review

Study	Country	Study Setting	Study type	Sampling	Size	Response rate	Population	Mean age/ range	Female (%)	Rural (%)	Proteinuria measurement	eGFR equation	Creatinine assessment method	HTN (%)	DM (%)	HIV (%)
Adebamowo 2016	Nigeria, Ghana, Kenya	outpatient	C/S	consecutive	4815	96	Diabetics, healthy controls	55	60	0	-	MDRD	Jaffe method	68.3	100	NR
Adedeji 2015	Nigeria	outpatient	C/S	consecutive	183	NR	HIV naïve	37.9	57.4	0		MDRD	Jaffe method	0	0	100
Afolabi 2009	Nigeria	outpatient	C/S	consecutive	250	NR	Family Practice patients	50.5	72.8	0	spot urine ACR	MDRD	Jaffe method	NR	6	NR
Akwanalo 2014	Kenya	community	C/S	consecutive	300	100	Hypertensive, healthy controls	58	65	100	-	MDRD	NR	100	56	NR
Anyabolu 2016	Nigeria	outpatient	C/S	consecutive	511	100	HIV naïve, healthy controls	38.8	72	NR	24-h proteinuria	MDRD	NR	NR	NR	100
Awobusuyi 2011	Nigeria	community	C/S	random	326	100	general population	43.5	58	0	urine dipstick test	CG	Jaffe method	46.6	0.9	NR
Ayodele 2011	Nigeria	community	C/S	consecutive	586	NR	general population	42.4	NR	0	urine dipstick test	-	-	25.9	4.1	NR
Ayokunle 2015	Nigeria	outpatient	C/S	consecutive	335	100	HIV naïve, healthy controls	40.3	56	NR	spot urine ACR	MDRD	Jaffe method	NR	NR	100
Bakshi 2014	Tanzania	outpatient	C/S	consecutive	49	NR	healthy adults	41	75.5	NR	-	MDRD	NR	0	0	0
Benghanem 2016	Morocco	community	C/S	stratified random	10524	85	general population	26-70	51.3	NR	urine dipstick test	MDRD	Jaffe method	21.9	16.8	NR
Booysen 2016	South Africa	community	C/S	random	1189	97	general population	44.1	65.1	NR	-	MDRD	Jaffe method	45	25.2	NR
Borkum 2014	South Africa	outpatient	C/S	consecutive	64	NR	HIV naïve	33	77	NR	spot urine ACR	MDRD	NR	NR	NR	100
Brenyah 2013	Ghana	outpatient	C/S	random	341	100	Diabetics	54.9	NR	0	urine dipstick test	-	-	NR	NR	NR
Cailhol 2011	Burundi	outpatient	C/S	consecutive	245	82	HIV	40.1	70.3	NR	urine dipstick test	CG, MDRD	NR	2.7	2	100
Egbi 2014	Nigeria	community	C/S	convenient	179	NR	general population	45.2	46.9	0	urine dipstick test	CG	NR	NR	NR	NR
Ekat 2013	Congo	outpatient	C/S	consecutive	562	74	HIV naïve	38.8	66.1	0	-	MDRD	Jaffe method	NR	NR	100
El-Tayeb 2010	Egypt	community	C/S	convenient	1260	100	general population	20.2	32.3	78.3	urine dipstick test	-	-	3.3	NR	NR
Emem 2008	Nigeria	outpatient	C/S	consecutive	400	NR	HIV	34.6	47.5	NR	urine dipstick test	CG	NR	NR	NR	100
Ene-Iordache 2016	Nigeria	KDDC database	C/S	consecutive	1912	NR	general population	44.4	63.4	NR	spot urine ACR	CKD-EPI	NR	NR	NR	NR
Ene-Iordache 2016	Egypt	KDDC database	C/S	consecutive	412	NR	high risk pop (at risk of or with CKD,	39.1	43.7	NR	spot urine ACR	CKD-EPI	NR	NR	NR	NR

							HTN, DM, CVD)									
Ephraim 2015	Ghana	outpatient	C/S	consecutive	208	87	Hypertensive, diabetics	60	71	0	spot urine ACR	CKD-EPI	Jaffe method	42.3	68.8	NR
Fana 2011	Zimbabwe	outpatient	C/S	consecutive	159	NR	HIV naïve	18+	NR	0	urine dipstick test	CG	NR	NR	NR	100
Feteh 2016	Cameroon	outpatient	C/S	consecutive	584	92	Diabetics	56.5	46.9	0	urine dipstick test	MDRD	Jaffe method	62.2	100	NR
Fiseha 2014	Ethiopia	outpatient	C/S	random	214	NR	Diabetics	45	42.5	NR	-	CG, MDRD	Jaffe method	52.8	100	NR
Glaser 2013	Malawi	outpatient	C/S	consecutive	363	100	HIV positive, healthy controls	31	48	NR	-	Cystatin C equation	NR	NR	NR	100
Janmohamed 2013	Tanzania	outpatient	C/S	consecutive	369	100	Diabetics	54	53.4	NR	urine dipstick test	CG	Jaffe method	57.5	100	NR
Jao 2011	7 countries	outpatient	C/S	consecutive	2495	NR	HIV naïve	30	70	NR	-	CG, MDRD	NR	NR	NR	100
Kamkuemah 2015	South Africa	outpatient	C/S	consecutive	1092	100	HIV naïve	34	62	0	-	CG	NR	NR	NR	100
Kaze 2013	Cameroon	outpatient	C/S	consecutive	104	NR	HIV naïve	35	68	0	urine dipstick test	MDRD	Jaffe method	NR	NR	100
Kaze 2015	Cameroon	community	C/S	multistage cluster	500	100	general population	45.3	46.6	0	urine dipstick test	CKD-EPI, CG, MDRD	Jaffe method	38.6	2.8	NR
Kaze 2015	Cameroon	community	C/S	multistage cluster	439	97.5	general population	47	57.9	72.9	spot urine ACR	CKD-EPI, CG, MDRD	Jaffe method	25.5	9.8	NR
Kaze 2016	Cameroon	outpatient	C/S	convenient	336	100	hypertensive	60.9	63.4	0	urine dipstick test	CKD-EPI, CG, MDRD	Jaffe method	100	18.5	3.6
Keegan 2011	Ghana	outpatient	C/S	consecutive	192	NR	HIV	NR	NR	NR	urine dipstick test	CG	NR	NR	NR	100
Longo 2012	DR Congo	outpatient	C/S	consecutive	300	NR	HIV	43	77	0	urine dipstick test	CG, MDRD	NR	NR	NR	100
Lucas 2010	Uganda	community	C/S	random	1806	97	HIV positive, healthy controls	28	61.3	NR	-	MDRD	Jaffe method	NR	NR	NR
Lunyer 2016	Uganda	community	C/S	convenient	141	NR	general population	18+	57	0	urine dipstick test	-	-	38	NR	NR
Mafundikwa 2007	Zimbabwe	outpatient	C/S	consecutive	75	NR	Diabetics	18+	NR	0	urine dipstick test	-	-	NR	100	NR
Makusidi 2013	Nigeria	community	C/S	random	535	100	general population	37	37.9	0	urine dipstick test	-	-	30.2	NR	NR
Masimango 2014	DR Congo	outpatient	C/S	consecutive	235	NR	HIV	40	73.2	100	urine dipstick test	-	-	30.6	20.4	100
Matsha 2013	South Africa	community	C/S	random	1202	96	general population	52.9	75.3	0	-	CKD-EPI, CG, MDRD	Jaffe method	NR	26.4	NR
Msango 2011	Tanzania	outpatient	C/S	consecutive	355	85	HIV naïve	36.1	65	0	urine dipstick test	CG, MDRD	NR	NR	NR	100
Mulenga 2008	Zambia	outpatient	cohort	consecutive	25249	71	HIV naïve	33.7	60.5	0	-	CG	NR	NR	NR	100

Nelissen 2014	Nigeria	community	C/S	random	1310	NR	Hypertensive, healthy controls	18+	64.8	100	-	MDRD	NR	100	6.8	NR
Ngassa 2015	South Africa	outpatient	C/S	consecutive	721	NR	Diabetics	57.2	62.9	0	-	MDRD	NR	79.2	100	NR
Obirikorang 2014	Ghana	outpatient	C/S	consecutive	163	NR	HIV	39.9	77.9	0	-	CKD-EPI, MDRD	NR	NR	NR	100
Odenigbo 2014	Nigeria	community	C/S	consecutive	170	77	general population	68.1	32.9	NR	-	MDRD	Jaffe method	NR	NR	NR
Odongo 2015	Uganda	outpatient	C/S	consecutive	361	98	HIV naïve	31.4	63.7	29.1	urine dipstick test	CKD-EPI	Jaffe method	0	0	100
Okafor 2016	Nigeria	outpatient	C/S	consecutive	383	NR	HIV naïve	35.4	58.5	0	spot urine ACR	MDRD	NR	0	0	100
Oladapo 2012	Nigeria	community	C/S	consecutive	415	NR	hypertensive	46.9	55.7	100	spot urine ACR	-	-	100	9.6	NR
Olanrewaju 2010	Nigeria	outpatient	C/S	consecutive	138	NR	hypertensive	43.2	45	0	urine dipstick test	-	-	100	NR	NR
Onodugo 2013	Nigeria	outpatient	C/S	consecutive	300	NR	HIV naïve	18+	65.3	0	-	CG	Jaffe method	0	0	100
Osafo 2011	Ghana	outpatient	C/S	consecutive	712	NR	hypertensive	59	78.7	0	spot urine ACR	MDRD	Jaffe method	100	14.7	NR
Peck 2016	Tanzania	community	C/S	multistage random	1043	72	general population	35.5	54.3	NR	-	CKD-EPI, MDRD	Jaffe method	17.3	0.9	8.4
Peck 2014	Tanzania	outpatient	C/S	consecutive	454	94	HIV positive, healthy controls	38	61.4	0	urine dipstick test	CKD-EPI	Jaffe method	16.3	0	67
Peer 2008	South Africa	outpatient	C/S	consecutive	403	NR	hypertensive	52	55	0	spot urine ACR	CG	NR	100	12	NR
Pruijm 2008	Seychelles	community	C/S	random	1218	80.2	general population	25-64	54.1	NR	spot urine ACR	MDRD	Jaffe method	23.7	36.1	NR
Rasmussen 2016	Zambia	outpatient	C/S	consecutive	160	NR	hypertensive	60.6	68.8	100	spot urine ACR	-	-	100	42.5	NR
Salako 2007	Nigeria	outpatient	C/S	consecutive	54	NR	hypertensive	59.1	46.3	0	urine dipstick test	CG	enzymatic method	100	1.9	NR
Sarfo 2013	Ghana	outpatient	C/S	consecutive	3137	78	HIV naïve	38	67	0	-	CKD-EPI, CG, MDRD	NR	NR	NR	100
Seck 2013	Senegal	community	C/S	random	402	53.2	general population	46.2	33.8	0	spot urine ACR	MDRD	NR	24.1	9.7	NR
Seck 2014	Senegal	community	C/S	multistage cluster	1036	99	general population	48	60	47	24-h proteinuria	MDRD	Jaffe method	39.1	12.7	NR
Stanifer 2015	Tanzania	community	C/S	multistage cluster	481	80	general population	45	74.4	23	urine dipstick test	MDRD	Jaffe method	28	12.7	NR
Struik 2011	Malawi	outpatient	C/S	consecutive	509	95	HIV naïve	34.3	66.5	0	-	CG	NR	NR	NR	100
Sumaili 2009	DR Congo	community	C/S	multistage cluster	500	99	general population	38.6	59	0	urine dipstick test	CG, MDRD	Jaffe method	27.6	11.7	NR
Sumaili 2009	DR Congo	outpatient	C/S	convenient	527	95	at risk population(HTN/DM/Obesity/HIV)	53.9	NR	0	dipstick (24HUP>300)	MDRD	Jaffe Method	58.2	54.5	43.4

Wensink 2015	South Africa	outpatient	C/S	random	903		HIV	40	69	100	-	CKD-EPI, MDRD	NR	23	4	100
Wools-Kaloustian 2007	Kenya	outpatient	C/S	consecutive	389	NR	HIV naïve	35	67.9	NR	urine dipstick test	CG, MDRD	NR	NR	NR	100
Wyatt 2011	Rwanda	outpatient	C/S	consecutive	865	NR	HIV positive, healthy controls	43	100	NR	urine dipstick test	CKD-EPI, CG, MDRD	Jaffe method	8.3	0.5	100
Wachukwu 2015	Nigeria	community	C/S	consecutive	259	NR	general population	28.3	47.9	0	urine dipstick test	CG	NR	20.8	4.3	NR
Oluyombo 2013	Nigeria	community	C/S	random multistage	454	NR	general population	45.8	56.6	NR	urine dipstick test	MDRD	NR	20.4	0.6	NR
Ekat 2012	Congo	outpatient	C/S	consecutive	562	NR	HIV naïve	38.8	66.6	0	-	MDRD	NR	NR	NR	100
Makulo 2010	DR Congo	community	C/S	random	229	NR	Diabetics, healthy controls	55.8	66.8	100	-	MDRD	NR	NR	100	NR
Ayodele 2007	Nigeria	outpatient	C/S	consecutive	147	NR	hypertensive	55.8	55.8	0	urine dipstick test	CG, MDRD	NR	100	NR	NR
Machingura 2016	Zimbabwe	outpatient	C/S	consecutive	344	98.9	Diabetics	57.6	72.7	0	spot urine ACR	CKD-EPI	Jaffe method	NR	100	NR
Akpan 2016	Nigeria	community	C/S	consecutive	502	NR	general population	38.5	70.6	0	urine dipstick test	MDRD	Jaffe method	30.2	5.8	NR
Divala 2016	Malawi	outpatient	C/S	consecutive	948	NR	HIV positive	43	71.7	49.6	urine dipstick test	-	-	23.7	4.1	100
Glaser 2016	Malawi	outpatient	C/S	consecutive	363	96	HIV positive, healthy controls	33.9	49	0	spot urine ACR	Cystatin C	Jaffe method	7.8	NR	100
Laurence 2016	South Africa	community	C/S	random	454	NR	general population	46.3	70.7	0	spot urine ACR	MDRD	NR	36.6	3.3	NR
Satman 2016	South Africa	outpatient	RCT	random	244	NR	Diabetics	NR	NR	NR	spot urine ACR	MDRD	Jaffe method	NR	100	NR
Mekuria 2016	Ethiopia	outpatient	C/S	consecutive	446	NR	HIV naïve	38.3	63	20.4	NA	CG	Jaffe method	26.3	17.5	100
Okafor 2016	Nigeria	community	C/S	consecutive	99	95.2	general population	50.7	73.6	100	urine dipstick test	CG	Jaffe method	17.2	8.1	NR
Stanifer 2016	Tanzania	community	C/S	stratified random cluster	45	79.4	Diabetics	45	66.7	23	urine dipstick test	MDRD	Jaffe method	53.4	100	NR
Fouad 2016	Egypt	community	C/S	consecutive	3000	NR	general population	22.8	54	0	spot urine ACR	CG	NR	NR	NR	NR
Ogola 2016	Kenya	outpatient	C/S	consecutive	385	NR	Diabetics	63.3	65.5	100	urine dipstick test	NR	NR	100	NR	NR
Owiredu 2013	Ghana	outpatient	C/S	consecutive	276	NR	HIV naïve	33.42	70	0	NA	CKD-EPI, CG, MDRD	Jaffe method	NR	NR	100
Beheiry 2015	Sudan	Outpatient	C/S	random	81	NR	hypertensive	NR	NR	NR	24-h proteinuria	NA	-	NR	NR	NR
Bolarinwa 2012	Nigeria	Outpatient	C/S	consecutive	72	NR	Sickle cell patients	24.3	72.2	0	spot urine ACR	CG	NR	NR	NR	NR

Abdu 2011	Nigeria	outpatient	C/S	consecutive	200	NR	Sickle cell patients	NR	50	0	urine dipstick test	-	-	NR	NR	NR
Cohen 2010	Malawi	outpatient	C/S	consecutive	522	85	Diabetics	53.2	NR	0	urine dipstick test	-	-	NR	100	NR
Ebong 2007	Cameroon	community	C/S	consecutive	482	NR	general population	NR	57.5	100	urine dipstick test	-	-	NR	NR	NR
Mpondo 2014	Tanzania	outpatient	C/S	consecutive	171	71.8	HIV naïve	38	70	0	urine dipstick test	CG, MDRD	Jaffe method	NR	NR	NR
Sumaili 2008	DR Congo	community	C/S	consecutive	3018	NR	general population	NR	NR	0	urine dipstick test	-	-	NR	NR	NR
Stohr 2008	Zimbabwe, Uganda	outpatient	RCT	random	3316	100	HIV naïve	NR	65	0	-	CG, MDRD	Jaffe method	NR	NR	100
Addo 2009	Ghana	community	C/S	consecutive	219	71.3	hypertensive	50.4	36	0	-	MDRD	Jaffe method	NR	NR	NR
Eastwood 2010	Ghana	community	C/S	cluster random	944	93.7	general population	54.7	62.4	100	-	CKD-EPI, CG, MDRD	Jaffe method	NR	NR	NR
Abu-Aisha 2009	Sudan	community	C/S	random	273	62.6	general population	34.3	50.9	0	urine dipstick test	CG, MDRD	NR	NR	NR	NR
Fatiu 2011	Nigeria	community	C/S	consecutive	286	NR	general population	49.5	90.2	100	urine dipstick test	-	-	37.7	NR	NR
Ulasi 2013	Nigeria	community	C/S	random	1941	89	general population	43.7	63.4	31.5	urine dipstick test	CKD-EPI	Jaffe method	26.1	5.9	NR

Table S3: Summary statistics for prevalence of CKD stages 1-5 in general populations

Group	Subgroup	N studies	Size	Prevalence (95% CI)	H (95% CI)	I^2 (95% CI)	p-heterogeneity	p-diff sub-groups	p-Egger test
Overall	-	22	23825	15.8 (12.1-19.9)	7.5 (6.8-8.2)	98.2 (98.0-99.0)	<0.001	<0.001	0.265
Equation	MDRD	14	16059	13.7 (10.2-17.6)	5.3 (4.6-6.2)	96.5 (95.0-97.0)	<0.001	-	0.029
	Cockcroft-Gault	8	5316	21.3 (9.9-35.5)	10.1(8.9-11.5)	99.0 (98.9-99.1)	<0.001	-	0.004
	CKD-EPI	5	4854	19.5 (13.8-25.9)	4.9 (3.8-6.5)	95.9 (93.0-98.0)	<0.001	-	0.789
	Cystatin C	1	247	8.5 (5.3-12.7)	-	-	-	-	-
Region	Overall	22	23825	15.8 (12.1-19.9)	7.5 (6.8-8.2)	98.2 (98.0-99.0)	<0.001	<0.001	0.265
	Central	3	1439	16.0 (7.3-27.2)	5.3 (3.7-7.7)	96.4 (93.0-98.0)	<0.001	-	0.035
	Eastern	3	881	14.4 (7.2-23.4)	3.3 (2.0-5.3)	90.7 (76.0-96.0)	<0.001	-	0.706
	Northern	3	13797	6.1 (3.6-9.3)	5.1 (3.5-7.5)	96.2 (92.0-98.0)	<0.001	-	0.409
	Southern	1	454	10.4 (7.7-13.5)	-	-	-	-	-
	Western	12	7254	19.8 (14.1-26.1)	6.1 (5.2-7.1)	97.3 (96.0-98.0)	<0.001	-	0.130
North vs Sub-Saharan		22	23825	15.8 (12.1-19.9)	7.5 (6.8-8.2)	98.2 (98.0-99.0)	<0.001	<0.001	0.265
	Sub-Saharan Africa	19	10028	17.7 (13.7-22.1)	5.4 (4.7-6.1)	96.5 (96.0-97.0)	<0.001	-	0.124
	North Africa	3	13797	6.1 (3.6-9.3)	5.1 (3.5-7.5)	96.2 (92.0-98.0)	<0.001	-	0.409
Publication year, median 2013	Overall	22	23825	15.8 (12.1-19.9)	7.5 (6.8-8.2)	98.2 (98.0-99.0)	<0.001	0.332	0.265
	Above median	15	19770	14.5 (10.3-19.3)	7.6 (6.8-8.5)	98.3 (98.0-99.0)	<0.001	-	0.826
	Below median	7	4055	18.6 (12.2-26.0)	5.4 (4.3-6.7)	96.5 (95.0-98.0)	<0.001	-	0.589
Study size (median, 446.5)	Overall	22	23825	15.8 (12.1-19.9)	7.5 (6.8-8.2)	98.2 (98.0-99.0)	<0.001	0.08	0.265
	Above median	11	21213	12.3 (8.5-16.6)	8.2 (7.2-9.3)	98.5 (98.0-99.0)	<0.001	-	0.570
	Below median	11	2612	20.0 (12.6-28.6)	5.2 (4.3-6.1)	96.2 (95.0-97.0)	<0.001	-	0.035
Age (median, 45 years)	Overall	22	23825	15.8 (12.1-19.9)	7.5 (6.8-8.2)	98.2 (98.0-99.0)	<0.001	0.814	0.265
	Above median	12	15318	15.3 (11.0-20.1)	5.9 (5.0-6.8)	97.1 (96.0-98.0)	<0.001	-	0.062

	Below median	10	8507	16.3 (9.4-24.8)	9.0 (7.9-10.2)	98.8	<0.001	-	0.004

Table S4: Summary statistics for prevalence of CKD stages 3-5 in general populations

Group	Subgroup	N studies	Size	Prevalence (95% CI)	H (95% CI)	I^2 (95% CI)	p-heterogeneity	p-diff sub-groups	p-Egger test
Overall	-	27	25929	4.6 (3.3- 6.1)	4.8 (4.3-5.3)	95.6 (94.0-96.0)	<0.001	<0.001	0.045
Equation	MDRD	21	22969	4.5 (3.0- 6.3)	5.1 (4.5-5.8)	96.2 (95.0-97.0)	<0.001	-	0.092
	Cockcroft-Gault	11	4927	11.8 (7.3-17.1)	5.2 (4.4-6.2)	96.4 (95.0-97.0)	<0.001	-	0.030
	CKD-EPI	7	6184	5.1 (3.1-7.6)	3.8 (2.9-5.0)	93.2 (88.0-96.0)	<0.001	-	0.110
	Cystatin C	1	247	2.4 (0.9-5.2)	-	-	-	-	-
Region	Overall	27	25929	4.6 (3.3- 6.1)	4.8 (4.3-5.3)	95.6 (94.0-96.0)	<0.001	<0.001	0.045
	Central	4	1587	6.8 (4.0-10.2)	2.4 (1.5-3.9)	82.8 (56.0-93.0)	0.001	-	0.349
	Eastern	7	3517	2.5 (0.6-5.5)	4.1 (3.2-5.3)	94.1 (90.0-96)	<0.001	-	0.944
	Northern	2	10797	2.6 (2.3-2.9)	7.1 (5.2-9.7)	99.0 (96.0-99.1)	<0.001	-	-
	Southern	2	2391	7.2 (6.1- 8.2)	7.1 (5.2-9.7)	99.0 (96.0-99.1)	<0.001	-	-
	Western	12	7637	5.3 (2.7-8.7)	5.4 (4.6-6.3)	96.6 (95.0-98.0)	<0.001	-	0.906
North vs Sub-Saharan	-	27	25929	4.6 (3.3- 6.1)	4.8 (4.3-5.3)	95.6 (94.0-96.0)	<0.001	0.004	0.045
	Sub-Saharan Africa	25	15132	4.8 (3.2-6.6)	4.6 (4.1-5.2)	95.3 (94.0-96.0)	<0.001	-	0.446
	North Africa	2	10797	2.6 (2.3-2.9)	7.1 (5.2-9.7)	99.0 (96.0-99.1)	<0.001	-	-
Publication year, median 2013	Overall	27	25929	4.6 (3.3- 6.1)	4.8 (4.3-5.3)	95.6 (94.0-96.0)	<0.001	0.015	0.045
	At or above median	19	21710	5.7 (4.0-7.8)	5.1 (4.5-5.8)	96.1 (95.0-97.0)	<0.001	-	0.006
	Below median	8	4219	2.4 (0.9-4.4)	3.6 (2.8-4.6)	92.1 (87.0-95.0)	<0.001	-	0.379
Study size (median, 402)	Overall	27	25929	4.6 (3.3- 6.1)	4.8 (4.3-5.3)	95.6 (94.0-96.0)	<0.001	0.555	0.045
	Above median	14	23524	4.2 (2.9-5.7)	4.9 (4.2-5.7)	95.8 (94.0-97.0)	<0.001	-	0.043
	Below median	13	2405	5.3 (1.7-10.7)	4.8 (4.1-5.7)	95.7 (94.0-97.0)	<0.001	-	0.003

Age (median, 43.7 years)	Overall	27	25929	4.6 (3.3- 6.1)	4.8 (4.3-5.3)	95.6 (94.0-96.0)	<0.001	0.013	0.045
	Above median	15	19177	6.4 (4.2-8.9)	5.6 (4.9-6.4)	96.8 (96.0-98.0)	<0.001	-	0.113
	Below median	12	6752	2.7 (1.3-4.6)	3.7 (3.1-4.6)	92.8 (89.0-95.0)	<0.001	-	0.256

Table S5: Summary statistics for prevalence of CKD stages 1-5 in high-risk populations

Group	Subgroup	N studies	Size	Prevalence (95% CI)	H (95% CI)	I^2 (95% CI)	p-heterogeneity	p-diff sub-groups	p-Egger test
Overall	Overall	21	5056	32.3 (23.4-41.8)	7.0 (6.3-7.7)	98.0 (97.9-98.1)	<0.001	-	0.005
Pathology	Overall	21	5056	32.3 (23.4-41.8)	7.0 (6.3-7.7)	98.0 (97.9-98.1)	<0.001	<0.001	0.005
	HIV	10	2007	27.3 (17.0-38.9)	5.5 (4.6-6.6)	96.7 (95.0-98.0)	<0.001	-	0.003
	HTN	6	2199	35.6 (27.9-43.7)	3.7 (2.8-5.0)	92.8 (87.0-96.0)	<0.001	-	0.331
	DM	4	778	32.6 (0.3-82.3)	13.9 (11.8-16.3)	99.5 (99.0-100)	<0.001	-	0.282
	Sickle cell	1	72	69.4 (57.5-79.8)	-	-	-	-	-
Equation	MDRD	11	3262	27.7 (17.1-39.7)	7.2 (6.3-8.3)	98.1 (97.0-99.0)	<0.001	-	0.005
	Cockcroft-Gault	6	1361	49.8 (28.8-70.8)	7.9 (6.6-9.5)	98.4 (98.0-99.0)	<0.001	-	0.039
	CKD-EPI	6	1234	34.7 (26.8-43.1)	2.9 (2.1-4.1)	88.0 (76.0-94.0)	<0.001	-	0.901
	Cystatin C	1	116	22.4 (15.2-31.1)	-	-	-	-	-
Region	Overall	21	5056	32.3 (23.4-41.8)	7.0 (6.3-7.7)	98.0 (97.9-98.1)	<0.001	<0.001	0.005
	Central	3	967	26.5 (7.5-51.6)	7.6 (5.7-10.2)	98.3 (97.0-99.0)	<0.001	-	0.195
	Eastern	6	1076	42.8 (21.8-65.3)	7.3 (6.0-8.8)	98.1 (97.0-99.0)	<0.001	-	0.003
	Northern	1	412	32.3 (27.8-37.0)	-	-	-	-	-
	Southern	2	308	2.7 (1.1-5.0)	9.7 (7.5-12.5)	99.5 (98.0-99.9)	<0.001	-	-
	Western	9	2293	36.6 (26.9-46.9)	4.8 (4.0-5.9)	95.7 (94.0-97.0)	<0.001	-	0.559
North vs Sub-Saharan	Overall	21	5056	32.3 (23.4-41.8)	7.0 (6.3-7.7)	98.0 (97.9-98.1)	<0.001	<0.001	0.005
	Sub-Saharan Africa	20		32.3 (22.8-42.5)	7.2 (6.5-7.9)	98.1 (98.0-98.4)	<0.001	-	0.008
	North Africa	1	412	32.3 (27.8-37.0)	-	-	-	-	-
Publication year, median 2014	Overall	21	5056	32.3 (23.4-41.8)	7.0 (6.3-7.7)	98.0 (97.9-98.1)	<0.001	0.233	0.005
	At or above median	13	2688	27.8 (18.4-38.3)	5.7 (4.9-6.6)	96.9 (96.0-98.0)	<0.001	-	0.001
	Below median	8	2368	39.9 (23.5-57.6)	8.4 (7.3-9.8)	98.6 (98.0-99.0)	<0.001	-	0.568
Study size (median, 192)	Overall	21	5056	32.3 (23.4-41.8)	7.0 (6.3-7.7)	98.0 (97.9-98.1)	<0.001	0.109	0.005

	At or above median	11	4022	39.3 (26.8-52.4)	8.5 (7.5-9.6)	98.6 (98.0-99.0)	<0.001	-	0.012
	Below median	10	1034	24.7 (14.0-37.2)	4.4 (3.6-5.3)	94.7 (92.0-96.0)	<0.001	-	<0.001
Age (median, 40.1 years)	Overall	21	5056	32.3 (23.4-41.8)	7.0 (6.3-7.7)	98.0 (97.9-98.1)	<0.001	0.466	0.005
	At or above median	12	3229	35.2 (22.6-49.0)	7.8 (6.9-8.9)	98.4 (98.0-99.0)	<0.001	-	0.025
	Below median	9	1827	28.4 (16.9-41.5)	5.8 (4.8-6.9)	97.0 (96.0-98.0)	<0.001	-	0.008

Table S6: Summary statistics for prevalence of CKD stages 3-5 in high-risk populations

**Group**	**Subgroup**	**N studies**	**Size**	**Prevalence (95% CI)**	**H (95% CI)**	**I^2 (95% CI)**	**p-heterogeneity**	**p-diff sub-groups**	**p- Egger test**
Overall	Overall	50	52353	13.3 (10.7-16.0)	7.8 (7.3-8.2)	98.3 (98.0-99.0)	<0.001	-	0.019
Pathology	Overall	50	52353	13.3 (10.7-16.0)	7.8 (7.3-8.2)	98.3 (98.0-99.0)	<0.001	<0.001	0.019
	HIV	28	44239	9.1 (6.6-11.9)	7.9 (7.3-8.6)	98.4 (98.0-99.0)	<0.001	-	0.018
	HTN	11	2971	17.9 (10.9-26.1)	5.3 (4.5-6.3)	96.5 (95.0-97.0)	<0.001	-	0.060
	DM	10	5071	22.0 (16.1-28.6)	4.9 (4.1-5.9)	95.8 (94.0-97.0)	<0.001	-	0.004
	Sickle cell	1	72	19.4 (11.1-30.5)	-	-	-	-	-
Equation	MDRD	31	21642	10.6 (7.6-13.9)	7.3 (6.8-8.0)	98.1 (98.0-98.3)	<0.001	-	<0.001
	Cockcroft-Gault	22	40610	16.2 (11.2-21.9)	11.7 (10.9-12.5)	99.3 (99.0-99.4)	<0.001	-	0.114
	CKD-EPI	11	6295	11.6 (6.2-18.3)	6.6 (5.7-7.7)	97.7 (97.0-98.0)	<0.001	-	0.203
	Cystatin C	1	116	4.3 (1.4-9.8)	-	-	-	-	-
Region	Overall	50	52353	13.3 (10.7-16.0)	7.8 (7.3-8.2)	98.3 (98.0-99.0)	<0.001	<0.001	0.019
	Central	7	2952	15.4 (8.4-24.0)	5.9 (4.8-7.2)	97.1 (96.0-98.0)	<0.001	-	0.002
	Eastern	21	34951	11.5 (8.0-15.6)	7.8 (7.1-8.5)	98.3 (98.0-99.0)	<0.001	-	0.035
	Northern	-	-	-	-	-	-	-	-
	Southern	4	3119	5.5 (0.9-13.6)	7.8 (6.2- 10.0)	98.4 (97.0-99.0)	0.002	-	0.125
	Western	17	8836	18.2 (13.3-23.7)	5.7 (5.1-6.5)	97.0 (96.0-98.0)	<0.001	-	0.835
Publication year, median 2013	Overall	50	52353	13.3 (10.7-16.0)	7.8 (7.3-8.2)	98.3 (98.0-99.0)	<0.001	0.324	0.019
	At or above median	29	14302	14.4 (11.0-18.2)	5.9 (5.4-6.5)	97.2 (97.0-98.0)	<0.001	-	<0.001
	Below median	21	38051	11.7 (8.0-16.0)	9.1 (8.4-9.9)	98.8 (98.6-99.1)	<0.001	-	0.025
Study size (median, 349.5)	Overall	50	52353	13.3 (10.7-16.0)	7.8 (7.3-8.2)	98.3 (98.0-99.0)	<0.001	0.330	0.019
	At or above median	25	47921	12.0 (8.9-15.6)	9.8 (9.1-10.5)	99.0 (98.9-99.1)	<0.001	-	0.060
	Below median	25	4432	14.7 (10.2-19.9)	4.6 (4.0-5.1)	95.2 (94.0-96.0)	<0.001	-	<0.001

Age (median, 40 years)	Overall	50	52353	13.3 (10.7-16.0)	7.8 (7.3-8.2)	98.3 (98.0-99.0)	<0.001	0.019	0.019
	At or above median	24	9535	17.2 (12.5-22.5)	6.3 (5.7-7.0)	97.5 (97.0-98.0)	<0.001	-	<0.001
	Below median	22	38861	10.3 (7.3-13.7)	8.0 (7.3-8.7)	98.4 (98.0-99.0)	<0.001	-	0.056

Item 4: Item 4: Supplementary figures

A: MDRD equation

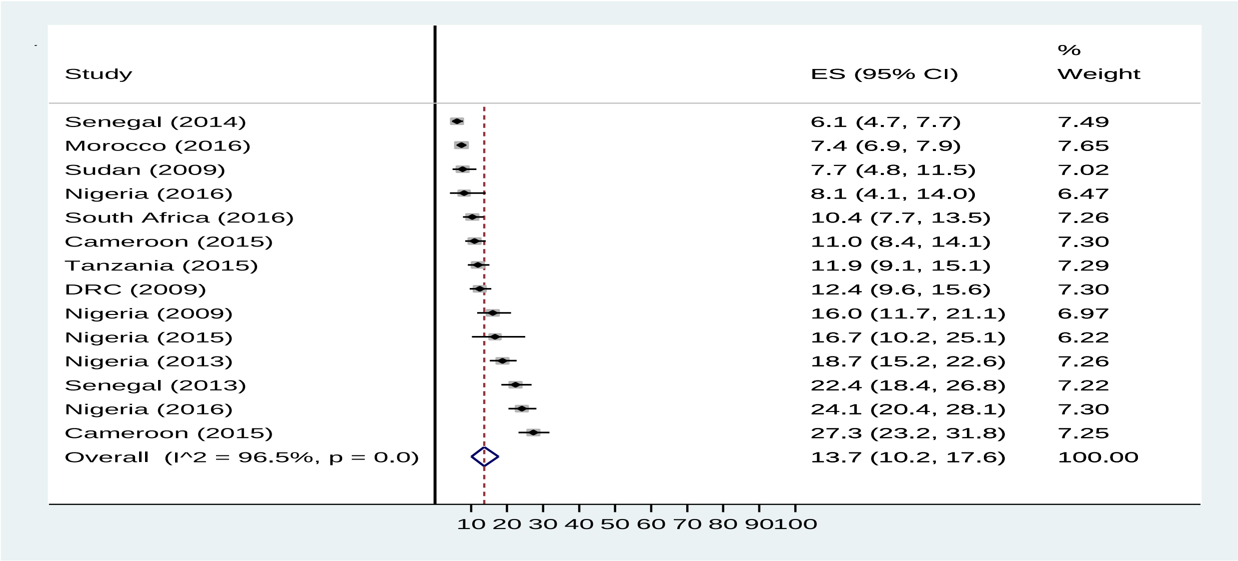

B: Cockcroft-Gault formula

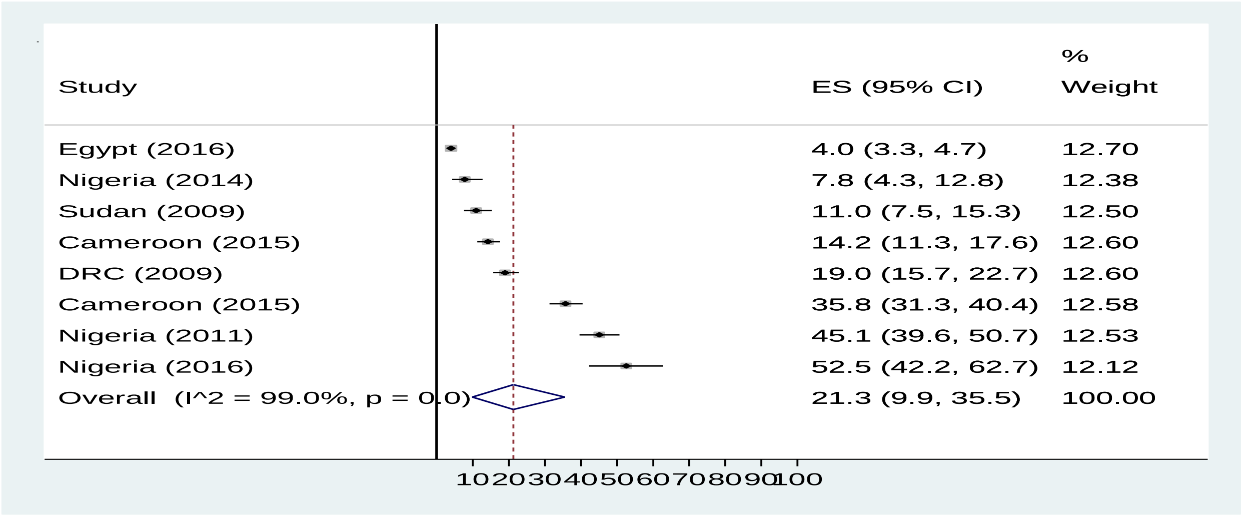

C: CKD-EPI formula

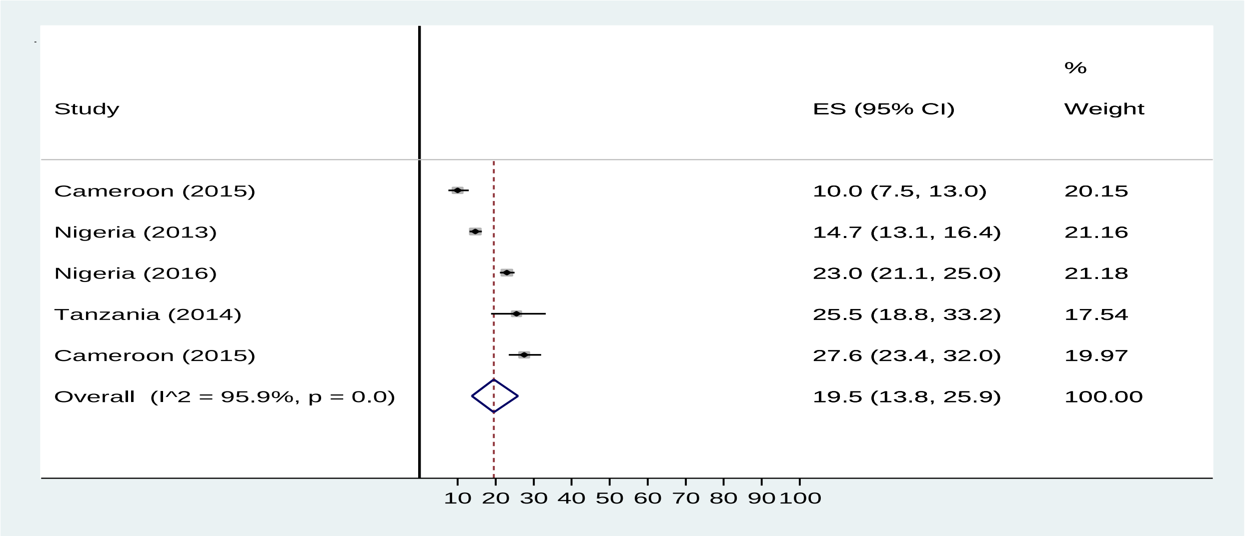

Figure S1: Prevalence of CKD in the general population of Africa according to eGFR equation. Figure panels are for the prevalence of CKD according to the MDRD equation (panel A), according to the Cockcroft-Gault formula (panel B), and according to the CKD-EPI equation (panel C). For each panel, the black boxes represent the effect estimates (prevalence) and the horizontal bars about are for the 95% confidence intervals (CIs). The diamond is for the pooled effect estimate and 95% CI and the dotted vertical line centered on the diamond has been added to assist visual interpretation.

A: MDRD equation

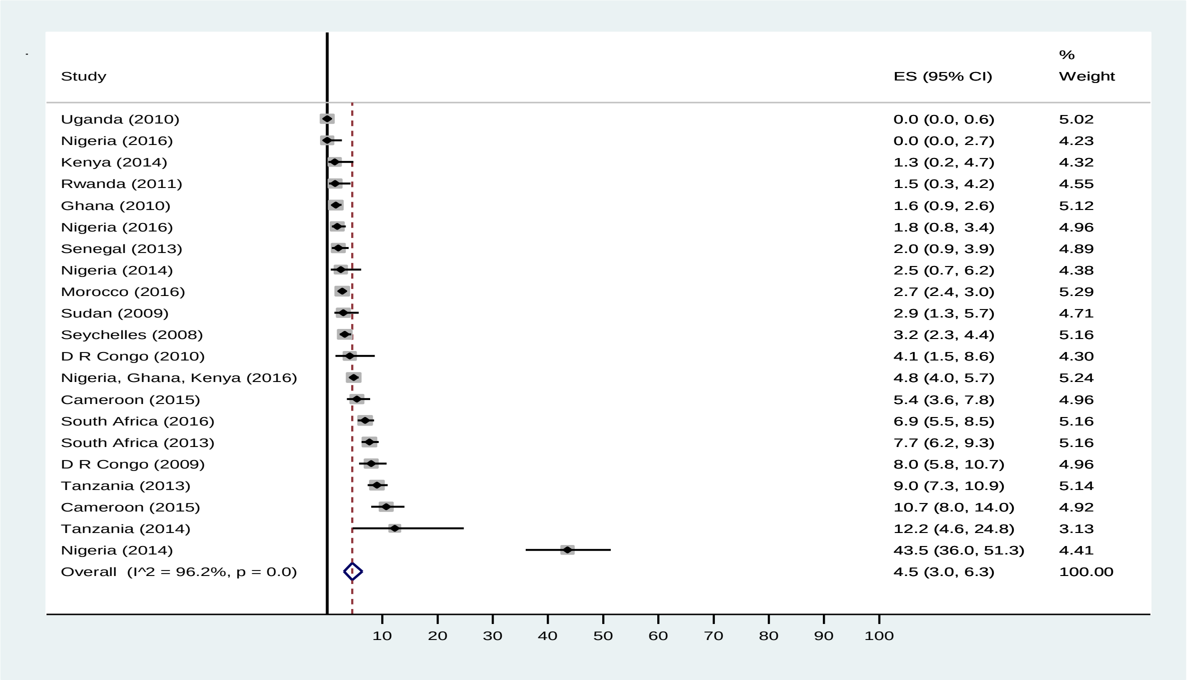

B: Cockcroft-Gault formula

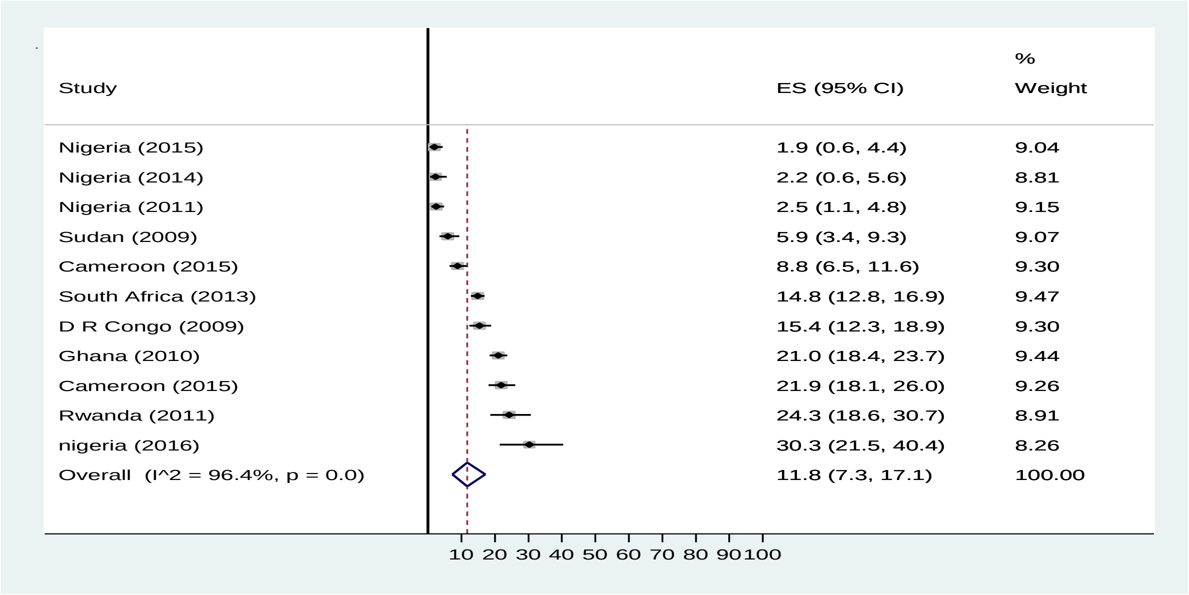

C: CKD-EPI equation

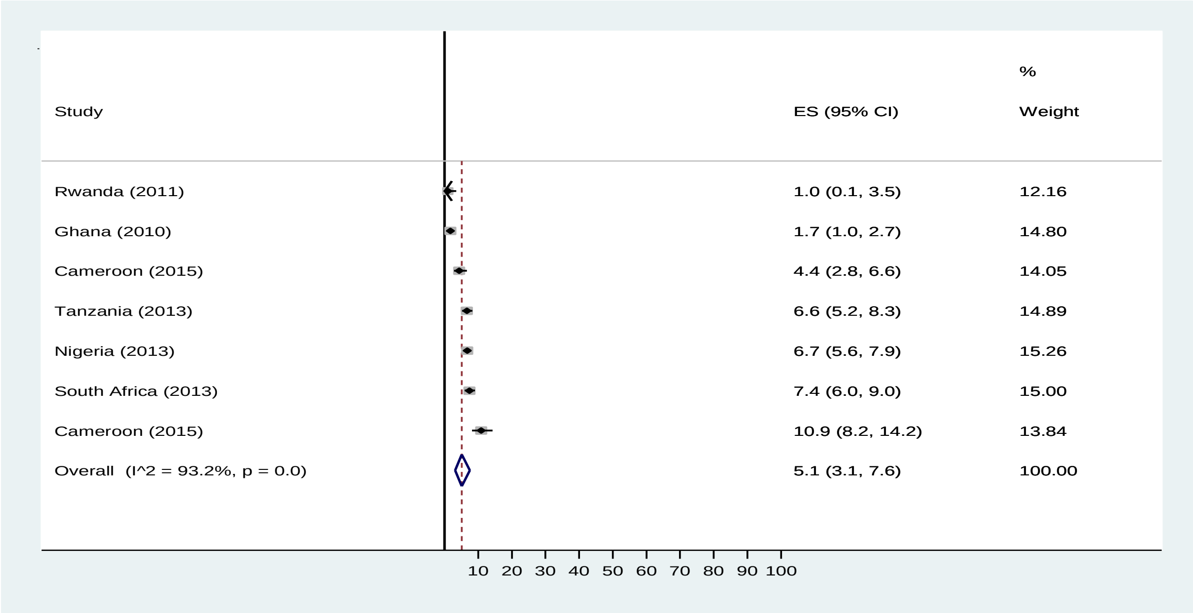

Figure S2: Prevalence of CKD stages 3 to 5 in the general population of Africa according to eGFR equation. Figure panels are for the prevalence of CKD stages 3-5 according to the MDRD equation (panel A), according to the Cockcroft-Gault formula (panel B), and according to the CKD-EPI equation (panel C). For each panel, the black boxes represent the effect estimates (prevalence) and the horizontal bars about are for the 95% confidence intervals (CIs). The diamond is for the pooled effect estimate and 95% CI and the dotted vertical line centered on the diamond has been added to assist visual interpretation.

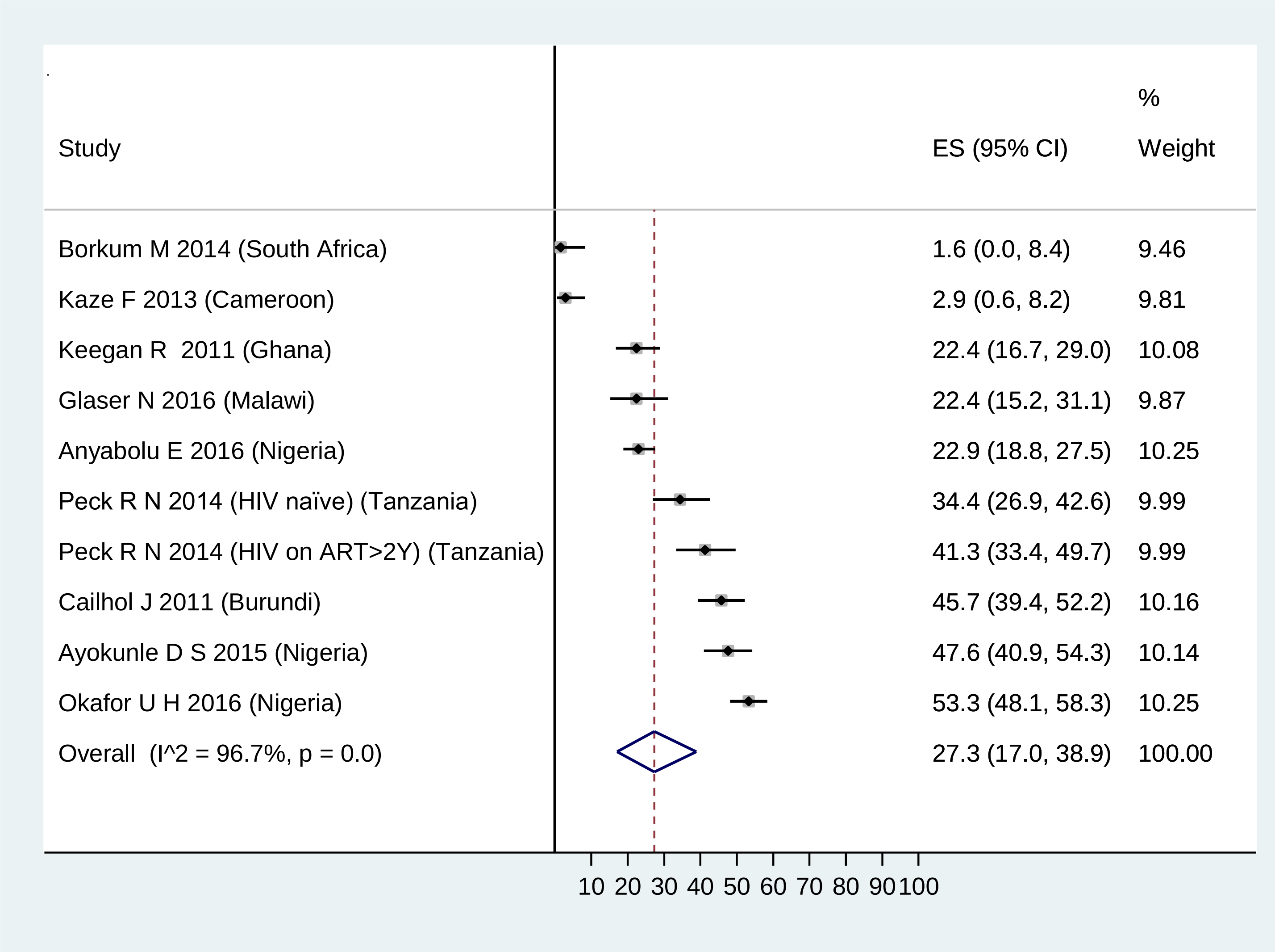

Figure S3: Prevalence of CKD in HIV-positive individuals living in Africa. Black boxes represent the effect estimates (prevalence) and the horizontal bars about are for the 95% confidence intervals (CIs). The diamond is for the pooled effect estimate and 95% CI and the dotted vertical line centered on the diamond has been added to assist visual interpretation.

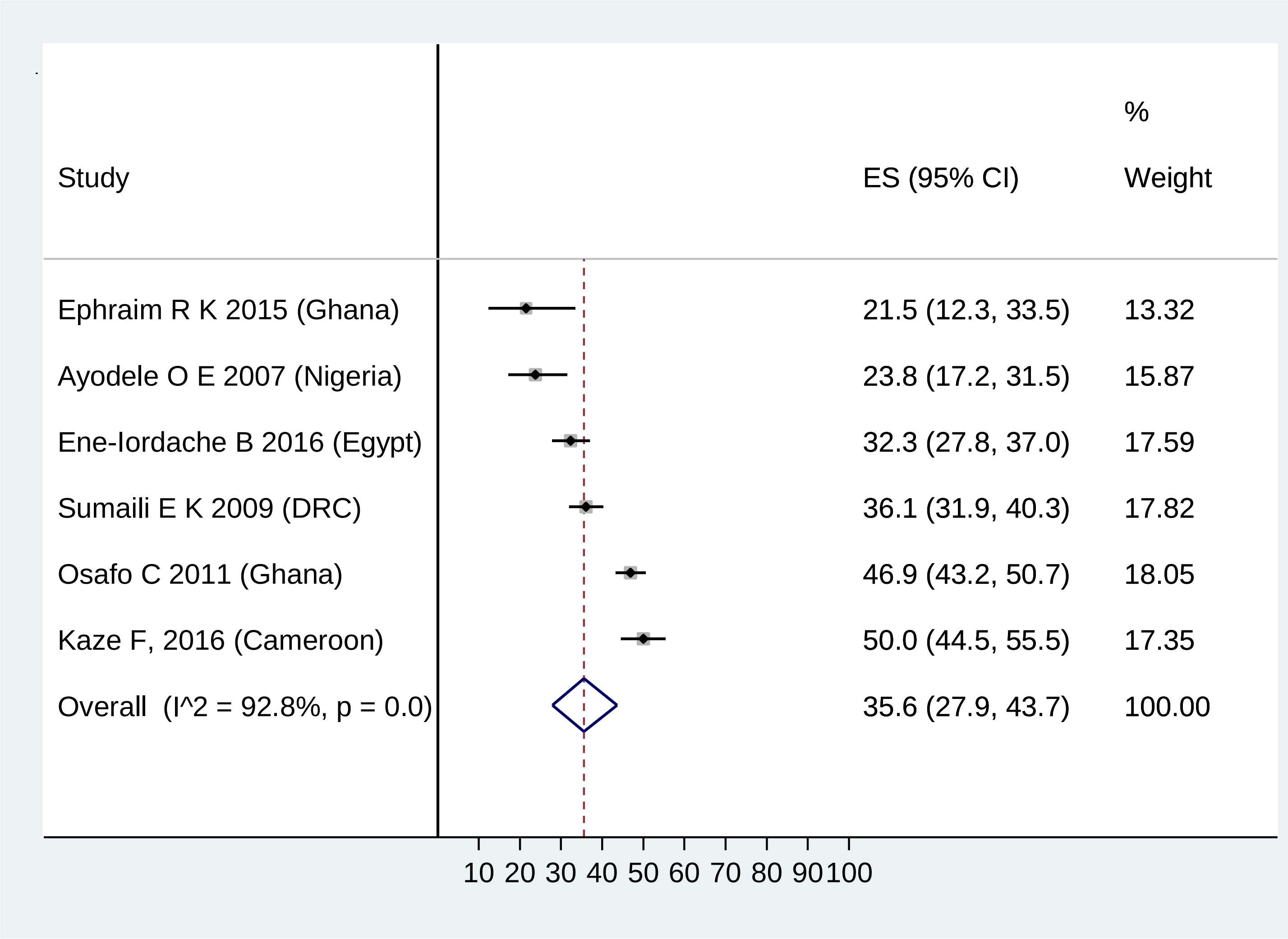

Figure S4: Prevalence of CKD in hypertensive individuals living in Africa. Black boxes represent the effect estimates (prevalence) and the horizontal bars about are for the 95% confidence intervals (CIs). The diamond is for the pooled effect estimate and 95% CI and the dotted vertical line centered on the diamond has been added to assist visual interpretation.

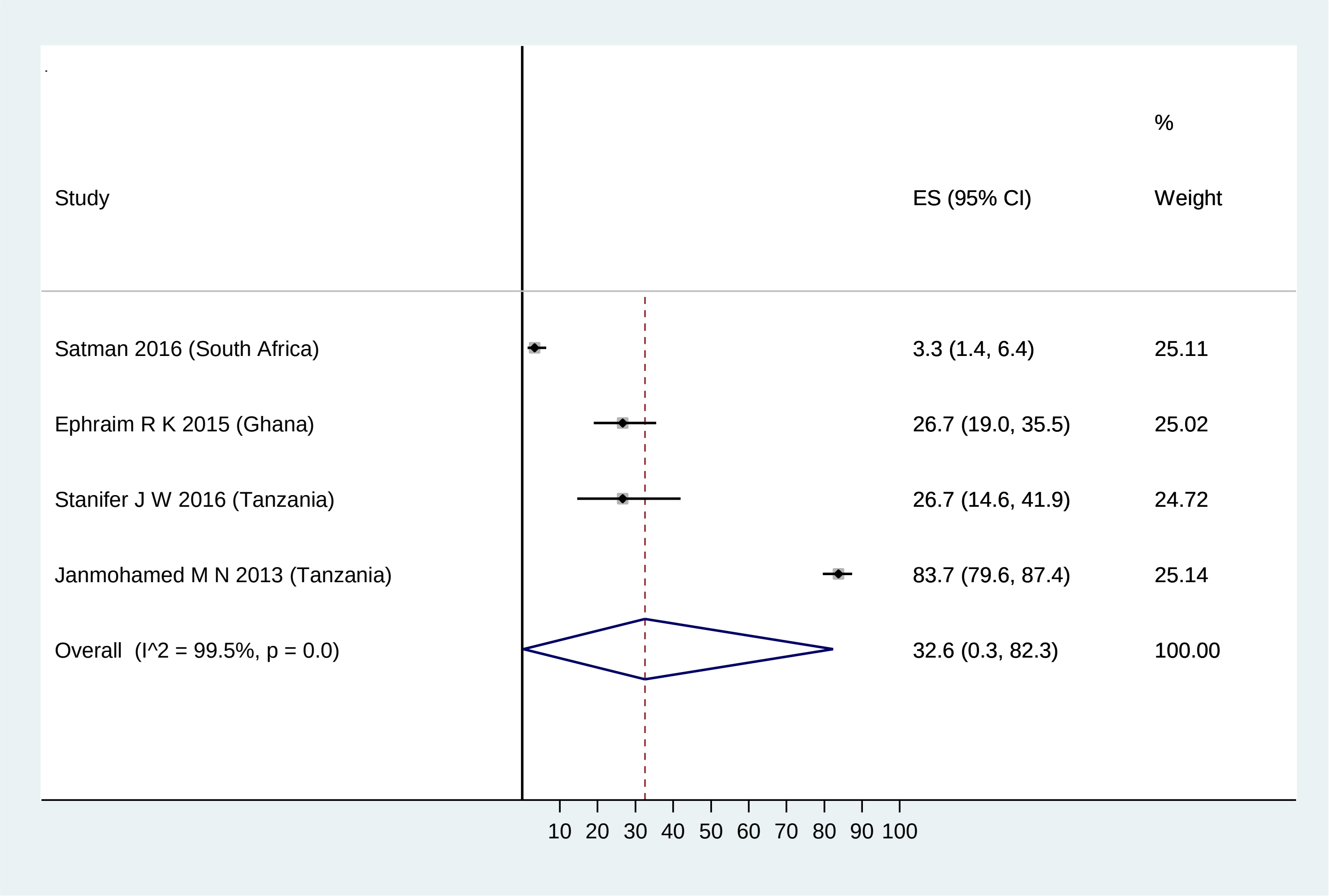

Figure S5: Prevalence of CKD in people with diabetes mellitus living in Africa. Black boxes represent the effect estimates (prevalence) and the horizontal bars about are for the 95% confidence intervals (CIs). The diamond is for the pooled effect estimate and 95% CI and the dotted vertical line centered on the diamond has been added to assist visual interpretation.

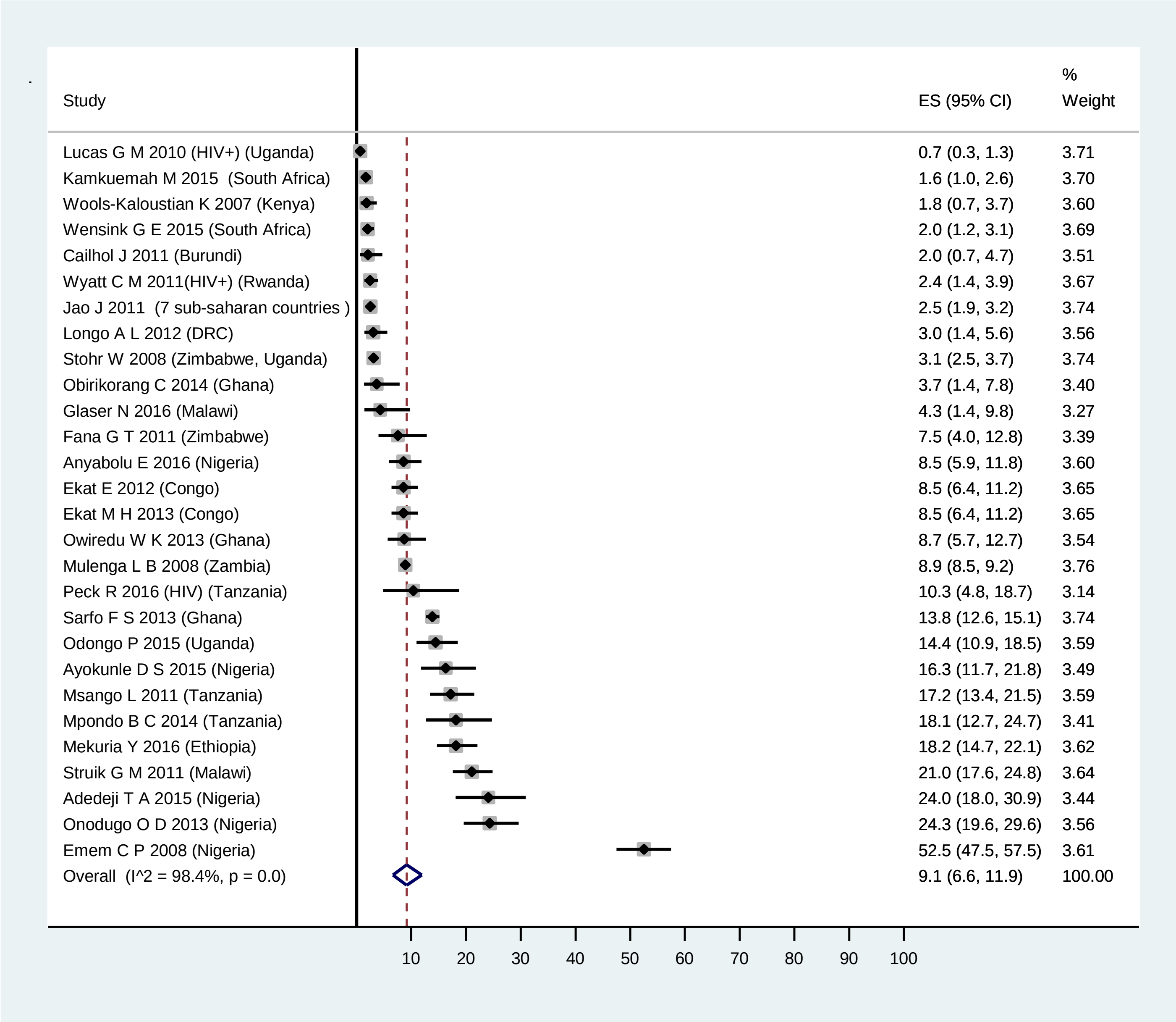

Figure S6: Prevalence of CKD stages 3 to 5 in HIV-positive individuals living in Africa. Black boxes represent the effect estimates (prevalence) and the horizontal bars about are for the 95% confidence intervals (CIs). The diamond is for the pooled effect estimate and 95% CI and the dotted vertical line centered on the diamond has been added to assist visual interpretation.

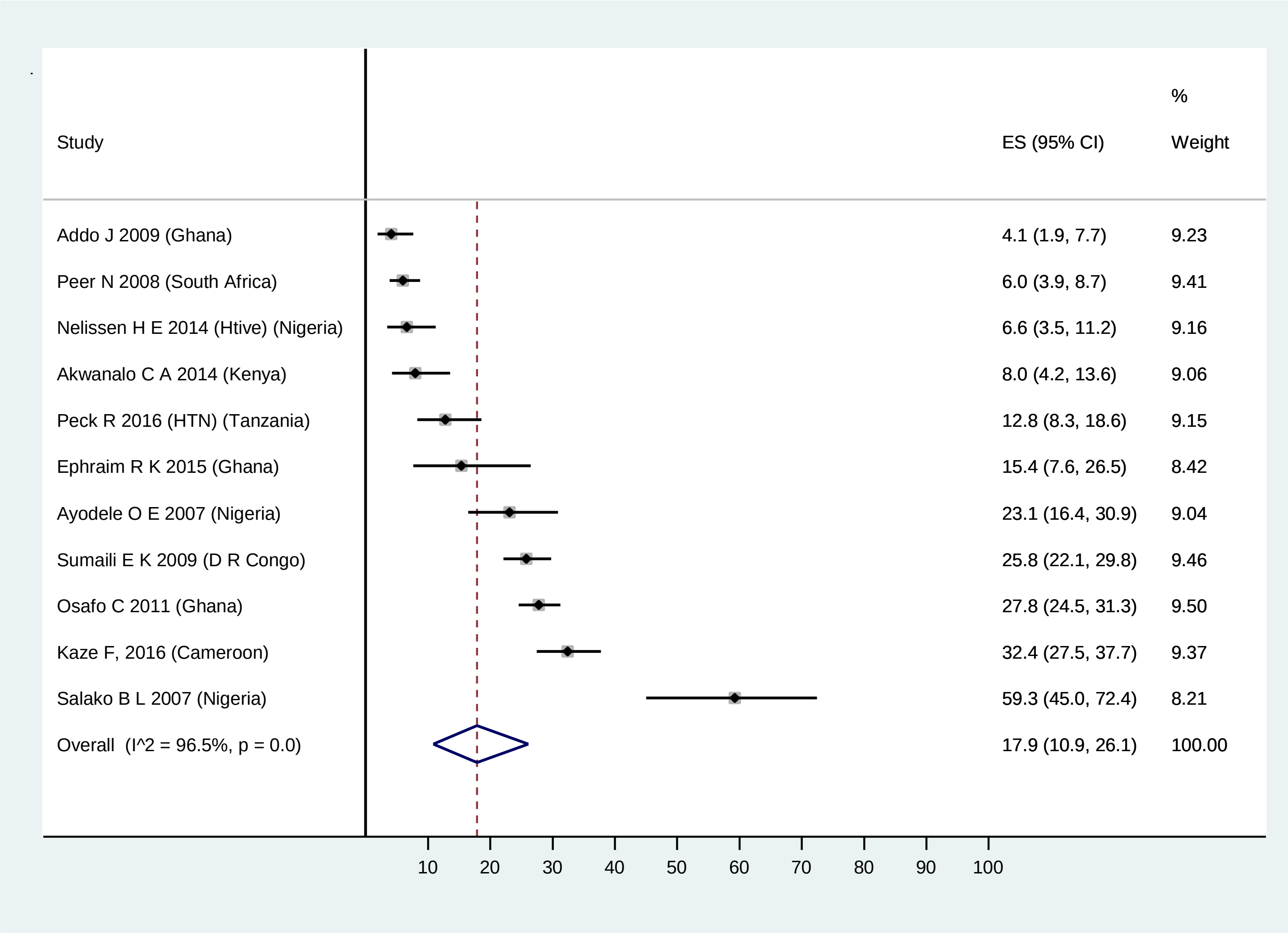

Figure S7: Prevalence of CKD stages 3 to 5 in hypertensive individuals living in Africa. Black boxes represent the effect estimates (prevalence) and the horizontal bars about are for the 95% confidence intervals (CIs). The diamond is for the pooled effect estimate and 95% CI and the dotted vertical line centered on the diamond has been added to assist visual interpretation.

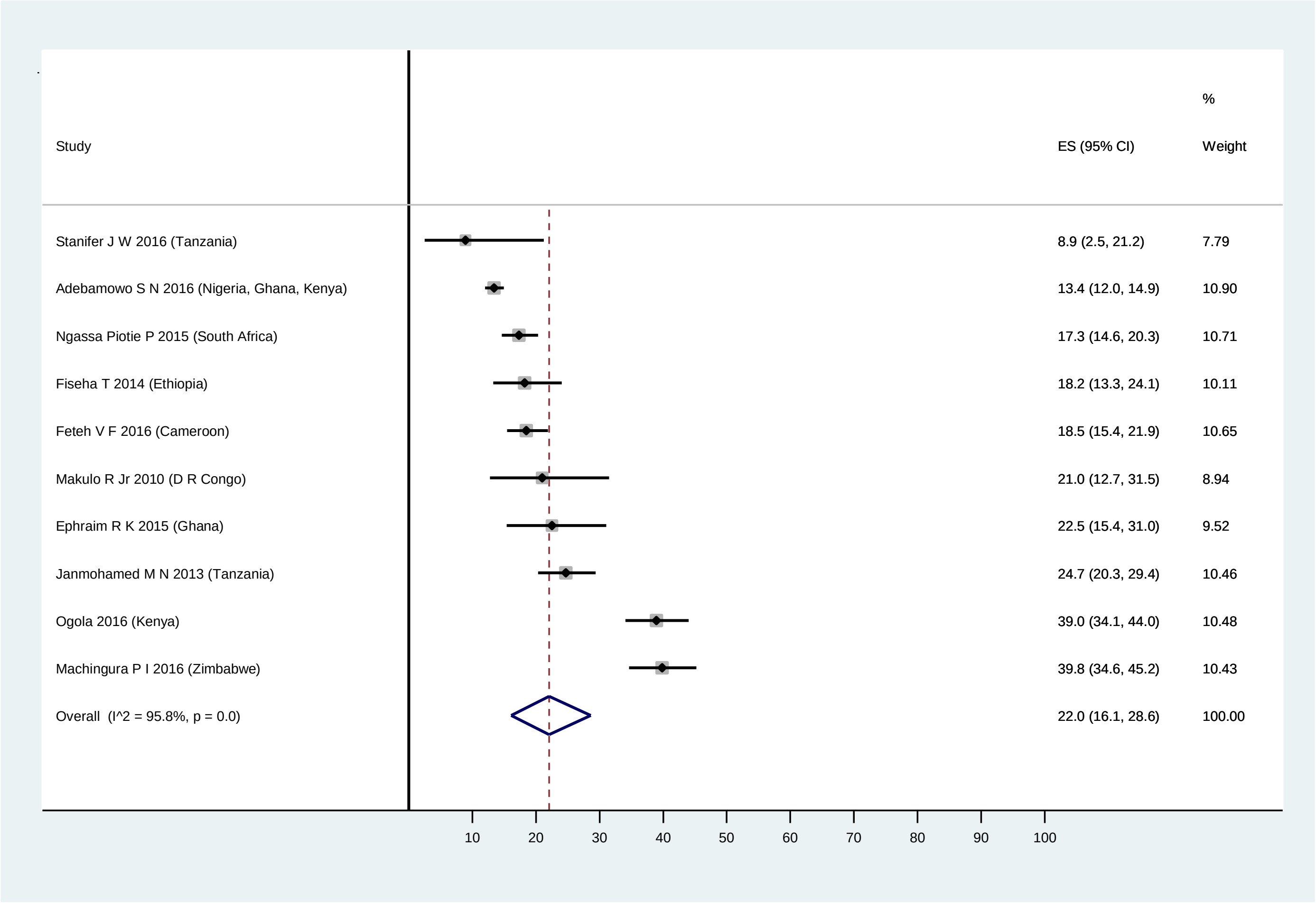

Figure S8: Prevalence of CKD stages 3 to 5 in people with diabetes mellitus living in Africa. Black boxes represent the effect estimates (prevalence) and the horizontal bars about are for the 95% confidence intervals (CIs). The diamond is for the pooled effect estimate and 95% CI and the dotted vertical line centered on the diamond has been added to assist visual interpretation.
